# Racial Discrimination and Administrative Burden in Access to Public Services

**DOI:** 10.1038/s41598-023-50936-1

**Published:** 2024-01-11

**Authors:** Elizabeth Bell, Sebastian Jilke

**Affiliations:** 1 The Lyndon B. Johnson School of Public Affairs, The University of Texas at Austin, Austin, USA; 2 The McCourt School of Public Policy, Georgetown University, Washington D.C., USA

**Keywords:** Psychology, Human behaviour

## Abstract

Equal access to public services is a foundational element of democratic societies. Yet, stark inequalities in access to public services persist, partially due to *bureaucratic discrimination,* or differential treatment by bureaucrats. This study investigates the causal mechanism of bureaucratic discrimination, arguing that racial discrimination can serve as a means of cream skimming, when there are economic incentives to prioritize easier-to-serve clientele. We predict that in the absence of information regarding prospective clients’ performance, group-level performance information will be imposed on racially minoritized individuals. We implemented a nationwide email correspondence audit experiment including all charter school principals in the U.S. (n = 5850). The findings show that Black email aliases faced significantly higher administrative burdens in trying to get access to charter schools than White email aliases when no performance signal was provided. However, when a direct signal of clients’ performance was introduced, the racial disparities diminished. Overall, these results provide evidence on the causal mechanism of bureaucratic discrimination as a means of cream-skimming.

Access to public services is a fundamental aspect of citizenship, and equal access to these services is a cornerstone of democratic societies^1,2^. However, evidence of racial and ethnic discrimination in access to public services, also referred to as *bureaucratic discrimination*, has been found in a wide range of studies^3–5^. One key finding of this literature is that employees of street-level organizations who directly interact with (potential) clients disproportionately increase administrative burdens (i.e., the hassles that make accessing public services more onerous) for members of racially and ethnically minoritized groups, which acts as a deterrent and thereby decreases equity in access to public services^6,7^.

The underlying mechanism explaining *why* we observe patterns of bureaucratic discrimination is unclear. The classical debate in economics of why people discriminate based on observed individual characteristics distinguishes between models of taste-based and statistical discrimination^8^. Meanwhile, social psychologists put forth models of stereotyping and implicit bias^9^. These approaches are stylized examples of why people treat members of racially minoritized groups differently; however, in the context of public service delivery, scholars lack a theoretical model explaining the underlying reasons for bureaucratic discrimination. This is important because the process of public service delivery is qualitatively different from making hiring decisions or choosing between potential tenants for an apartment. While public services ought to be delivered *sine ira et studio*–without hatred or passion, as Max Weber puts it^10^–the distinct institutional characteristics of public service organizations shape discriminating agents’ incentive structures and thus their motive to discriminate. Specifically, we argue that organizations that operate within public service markets may be incentivized to treat potential clients differentially because of the potential economic consequences^11–13^.

To inform our theoretical predictions, we draw from models of statistical discrimination which posit that unequal treatment is the result of an ecological fallacy in which the discriminating agent applies group-level knowledge as a stereotype towards members of specific groups to compensate for missing individual-level information^14,15^. (While standard models of statistical discrimination assume that discriminating agents have full knowledge about relevant statistical information at the group level, stereotypes about group differences may lead to distortions of accurate statistical beliefs^16^. However, the underlying process of accurate or inaccurate statistical discrimination is substantively the same: group-level information and beliefs are used as stereotypes against individuals.) Missing information may include indicators of future productivity in labor markets, or bureaucratic success criteria in the case of public service delivery^17^. Bureaucratic success criteria comprise client characteristics that are associated with organizational success–such as employment agencies focusing on clients with the highest chance of gaining employment, or college pipeline programs who prioritize students with the highest chances of getting into college^18^. These criteria often have economic consequences such as funding, organizational expenses, or work effort. Thus, Michael Lipsky in his seminal work on street-level bureaucracy argues that frontline employees tend to prioritize clients who are most likely to succeed, and disregard those who are perceived as costly to the organization^18^. Indeed, evidence for cream skimming, defined as the intentional selection, or avoidance, of certain clients into public services or programs, has been found in areas with high-powered performance management regimes^19^ or within marketized public services^20^, where economic consequences encourage discriminatory practices.

When engaging in cream-skimming, bureaucratic success criteria need to be directly observable. However, this is often not the case because of two reasons: (i) they are impending and/or (ii) historical proxies are typically not shared with frontline staff. Thus, when direct information about bureaucratic success criteria are not available, discriminating agents use imperfect information–observable client characteristics that are correlated with the respective criteria in question. We argue that this leads to bureaucratic discrimination. In this study, we test whether this generalized mechanism of bureaucratic discrimination predicts unequal treatment in access to public services that are delivered within public service markets. This is a substantial addition to the literature on bureaucratic discrimination and administrative burden in citizen-state interactions. It also has normative implications for market-based models of public service delivery arrangements because it implies that the economic incentive structures within public service delivery organizations may induce discriminatory practices.

The charter school sector, where education–a key public service–is provided under a market-based model, offers a unique opportunity to test our theoretical prediction. Charter schools need to attract students and competitive funding to survive economically^21^, placing a monetary incentive on schools to focus on students who are less costly and have high academic achievement^22^. This provides a motive to prioritize students who are easier-to-serve and are projected to perform well academically in the future. Concerns over this type of cream skimming in the charter sector have been voiced repeatedly^23^, and some evidence suggests that charters prioritize students who are performing well academically^24,25^, while others found little evidence to this effect^21,26^. In a pre-study experiment among 490 charter school principals, we found that students who perform well academically are more likely to be prioritized for admission (see SI Appendix S6). Specifically, in the context of a conjoint-based admission task, principals are about 10 percentage points more likely to say they would admit a child who tests above state average in math as compared to a child who tests well below state average.

Unlike our conjoint-based admissions task, test score information is not always observable, leaving frontline employees working in public service delivery organizations with imperfect information as they attempt to prioritize students with the highest future test scores^22^. Here, race may serve as a shorthand for being a potentially costly client because it is correlated with numerous bureaucratic success criteria such as standardized test scores, English language proficiency, disciplinary records, and special needs status^26^. Taking the well-established Black-White achievement gap in standardized testing in the U.S. as an example^27^, African-American students compared to White students perform less well academically in terms of their literacy, numeracy and writing skills. But this may not be true for a specific student; here, group-level statistics may be used as a stereotype towards individual students when direct information (like standardized test scores or GPAs) are unavailable. The key empirical implication is that bureaucratic discrimination may be, in part, a response to missing information about bureaucratic success criteria. Indeed, when contacted prior to applying to either enroll into the school or its respective lottery, schools have no access to direct information about students’ academic performance. Instead, school officials may use imperfect signals such as race to weed-out undesirable students by making the application process more onerous or burdensome.

Street-level managers of charter schools can increase administrative burdens of prospective clients along different dimensions. The administrative burden framework distinguishes between three different types of costs that clients who are interacting with public service delivery organizations can encounter: learning, compliance or psychological costs^28^. Learning costs include the difficulty that people face in trying to find, access, and understand information required to access public services^29^. This includes learning where and how to apply to a charter school or a district lottery. Compliance costs are the bureaucratic rules and paperwork required for receiving said services that determine whether applicants are eligible. This includes mean-testing for welfare programs, or selective entrance requirements for schools. Lastly, psychological costs represent the stress and stigma, including loss of autonomy, that applicants face in the process of applying, or while receiving services. A common example of psychological costs is the stigmatization that recipients of means-tested benefits sometimes face from other members of the public, but psychological costs can also arise from interactions with unfriendly or unhelpful frontline workers. What all of these different costs have in common is that they can be altered by employees of street-level organizations and those who directly interact with clients–like the staff that answers information inquiries. These frontline workers can move towards clients^30^ and provide them with extra information and support. But they can also give applicants incomplete information, or simply not respond to requests at all^31^. While altering administrative burdens does not lead to discrimination per se, doing so disproportionately for clients with certain observed characteristics leads to bureaucratic discrimination.

Audit studies of public and charter schools have tested for some types of bureaucratic discrimination but have not provided a clean test of the mechanism we propose that may be driving discrimination. First, Bergman et al. conducted an audit study with a sample of U.S. public and charter schools and found evidence of discrimination against students with disabilities, students with lower test scores, and Hispanic students^32^. The study’s results emphasize the distinct role of test scores for charter schools in selecting students. However, it does not test whether these bureaucratic success criteria changed the likelihood of discrimination. Second, in the context of public schools in Denmark, Olsen et al., test whether positive or negative performance information about a Muslim student changes the likelihood that they are discriminated against in the responses of public school officials in Denmark^33^. They find that discrimination against Muslim students persists regardless of good or bad student grades; however, they did not include an experimental condition that tests for discrimination in the *absence* of any information on student academic achievement. In sum, while these two studies examine whether there is evidence of discrimination, they did not test whether information, relative to no information on bureaucratic success mitigates racial/ethnic discrimination. This is an important distinction because our theoretical framework emphasizes the availability of student information, and not its valence, as a key driver of discriminatory practices in the context of cream skimming. In another recent audit study of a sample of U.S. public and charter schools, Oberfield and Incantalupo test whether positive or negative performance information mitigates anti-Black discrimination relative to a no information condition^25^. However, the authors do not specifically reference test scores and grades as key bureaucratic success criteria; instead, the study’s treatment conditions highlight student conduct and implies that parents want to place their kids in advanced placement courses, or that they need extra help because they struggle with school. These factors are not as directly linked to race as test scores. As a result, the study’s findings are in contrast to our theoretical predictions, which is likely a result of not directly referencing the most important bureaucratic success criteria that is linked to race: standardized test scores. We build on these studies by directly testing whether the presence of information regarding the performance on standardized tests mitigates anti-Black discrimination on the full population of U.S. charter schools.

In the remainder of this article, we test whether street-level managers will increase administrative burdens for racially minoritized clients in the absence of any signal of bureaucratic success criteria in a nationwide email correspondence audit experiment including all charter school principals in the U.S. (n = 5850), which we identified using the National Center for Education Statistics directory. Specifically, we argue that student performance on standardized tests serves as the most important bureaucratic success criteria for charter schools. The mere absence of such information will increase the probability that school administrators use imperfect information, such as race, as a proxy to determine whether it is worth investing time into recruiting the student to enroll in their school. This process, we argue, leads to racial discrimination in access to charter schools.

## Results

This study randomized two different factors (i.e., the race of putative senders and a signal of academic performance) within information requests sent to charter school principals (see “Materials and Methods” section for more detail). We begin by presenting the summary statistics for each administrative burden outcome measure in Table 1, broken down by whether charter school principals were responding to an email with a randomly assigned White or Black email alias and an email with or without the signal of bureaucratic success (i.e., whether it was mentioned that the student had good grades and test scores–see “Materials and Methods” for more details). Of the 10,880 email requests we sent to charter school principals, 4203 received responses within our pre-registered analytical timeframe (4 weeks). Without accounting for failed contacts, this gives us an overall 38.6% response rate, and when we include only successfully delivered emails (9,897 emails), our response rate is 42.5%.

**Table 1:** Summary Statistics for Primary and Secondary Outcomes.

Variable	Black	White	Signal	No signal
Emails sent	5301*	5579	5494	5385
Failed contact: incorrect address/spam filter	459	524	498	485
Learning costs (dropping failed contact)				
Response	1938 (40.0%)	2265 (44.8%)	2006 (40.1%)	2197 (44.8%)
Answered how to apply	1328 (27.4%)	1594 (31.5%)	1387 (27.8%)	1535 (31.3%)
Compliance costs (dropping failed contact)				
Follow up questions asked	125 (2.6%)	100 (1.9%)	113 (2.3%)	112 (2.3%)
Answered yes anyone can apply	813 (16.8%)	1000 (19.8%)	833 (16.7%)	980 (20.0%)
Psychological costs (dropping failed contact)				
Greeting included	1648 (34.1%)	1966 (38.9%)	1731 (34.7%)	1883 (38.4%)
Salutation included	1210 (25.0%)	1386 (27.4%)	1240 (24.8%)	1356 (27.7%)

This table presents the descriptive statistics for each of our outcome measures of administrative burden (separated into learning, compliance, and psychological costs), across the groups randomly assigned a Black vs. a White email alias. *The sample size is slightly smaller for Black relative to White emails sent because 1 of the 10 email accounts with a Black alias malfunctioned in the second round. To address this issue, we drop the data for this email account in Round 2.

For each outcome, across the administrative burden cost categories, we find disparities between Black and White email aliases–by this we mean emails that were sent by putative Black or White senders, respectively (see “Materials and Methods” for more detail). For learning costs, the response rate for White email aliases is approximately 4.8 percentage points higher than Black email aliases (44.8% versus 40.0%), and White email aliases are approximately 4.1 percentage points more likely to get a response to the question of how to apply (31.5% versus 27.4%). Moreover, Black email aliases were more likely to face increased compliance costs–Black email aliases were less likely to be told that “anyone can apply” (16.8% versus 19.8% for White emails) and more likely to face follow-up questions and verification requests (2.6% versus 1.9% for White emails). To demonstrate what these disparities in compliance costs look like in practice, we provide anonymized example email responses in SI Appendix section S5. Finally, Black email aliases were 4.8 percentage points less likely to receive a greeting and 2.4 percentage points less likely to receive a salutation. We classify greetings and salutations as psychological costs in line with prior audit studies, as greetings and salutations are an objective way to measure the friendliness of the reply^33^.

**Figure 1:**
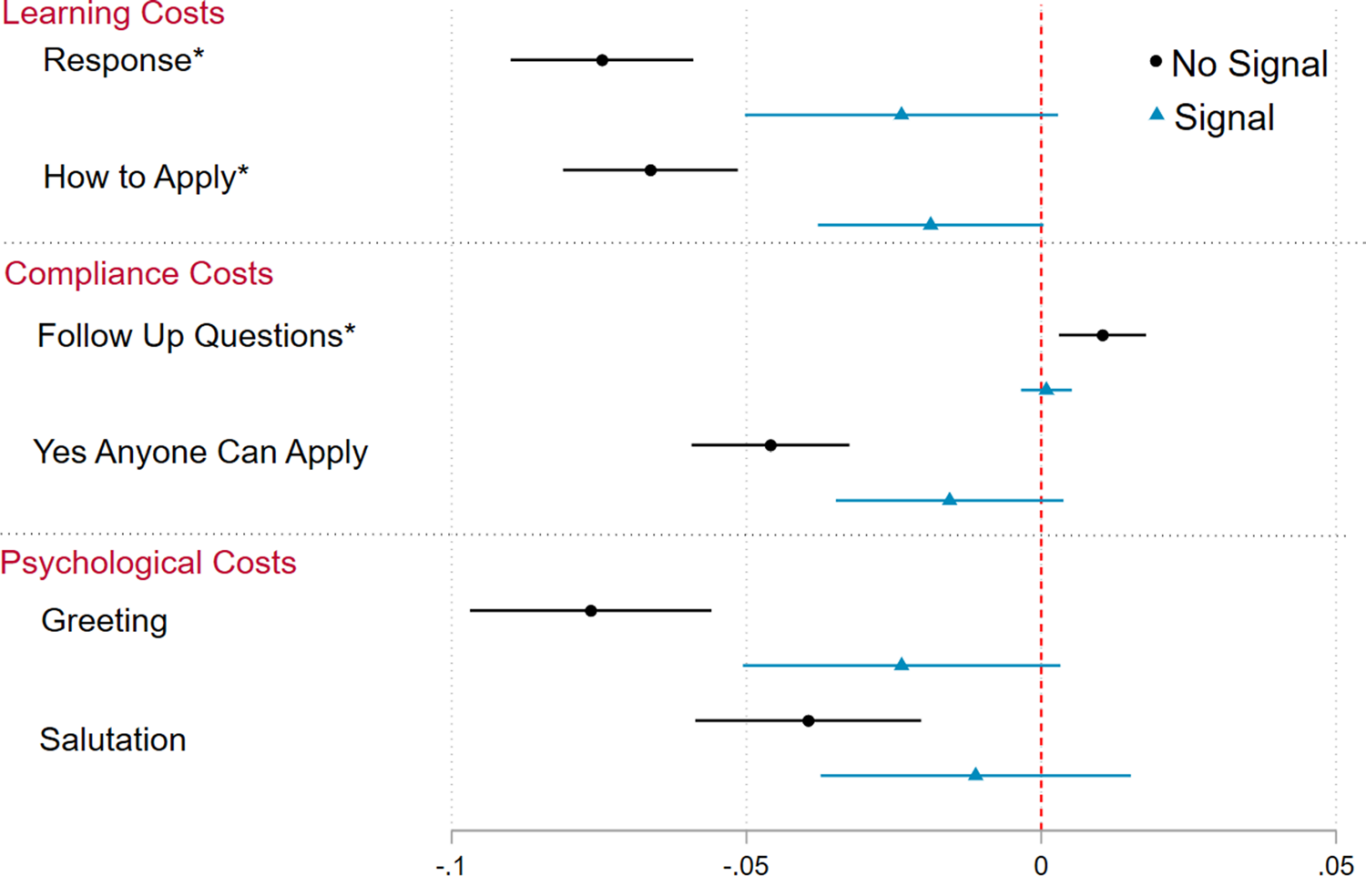
Treatment effect estimates of being randomly assigned a Black email alias, relative to a White email alias, across signal conditions (see “Materials and Methods” section for more detail on the experimental materials and randomization procedure). Each outcome reflects a learning, psychological, or compliance cost that the hypothetical parent would face based on the response, or non-response, of the charter school official. Point estimates and 95% confidence intervals estimated via OLS regression with state fixed effects to increase statistical precision and robust standard errors clustered by management organization to account for the possibility that umbrella organization charter schools have standardized responses to information requests.

Figure 1 presents OLS regression results with state fixed effects and standard errors clustered by management organization and by the school identifier (We cluster by school identifier to account for the multiple rounds of emails sent, and we also cluster by management organization to account for the possibility of a standardized response among schools with the same management organization. The model results are presented in SI Appendix section S2.1. We also estimate variants of this model where we include fixed effects for randomization blocks (state and management organization type) and the results are robust to these alternative specifications (see SI Appendix section S2.2).) We exclude all instances of bounced emails or failed contact in the estimation of these results and we use a pre-registered Benjamini–Hochberg multiple comparisons adjustment^34^ with a five percent false discovery rate for our pre-registered primary outcomes (marked with an asterisk) to account for multiple testing of our hypotheses.

The results in Fig. 1 provide strong support for our theoretical hypothesis across all administrative burden cost categories–Black email aliases face significantly higher learning, compliance, and psychological costs when there is no direct signal of a bureaucratic success criteria. However, racial disparities in administrative burden diminish once a signal of bureaucratic success is included.

Beginning with learning costs, the results in Fig. 1 demonstrate that Black email aliases were 7.4 percentage points less likely to receive a response when there was no signal (*p* < 0.000) but only 2.4 percentage points less likely to receive a response when there was a signal of bureaucratic success criteria (*p* = 0.080). In terms of magnitude, the treatment effect in the no signal condition reflects a 15.4 percent decrease in the likelihood of getting a response as a function of being randomly assigned a Black email alias, whereas the effect of race in the signal condition reflects a statistically insignificant 5.7 percent change. When we interact the two treatment dimensions (see SI Appendix S2.2), we find that the treatment effects of race in the signal and no signal conditions are statistically distinguishable from one another (*p* = 0.005). For our second measure of learning costs, we find that Black email aliases are 6.6 percentage points less likely to receive information on how to apply when there is no signal (*p* = 0.008), which translates to a 19.2 percent decrease. However, when there is a signal of bureaucratic success criteria, Black email aliases are only 1.9 percentage points less likely to receive information on how to apply (*p* = 0.055). When we test the interaction between treatment groups on the likelihood of receiving information on how to apply, the treatment effect estimates for race across signal conditions are significantly different from one another (*p* = 0.000).

Moving to compliance costs, we find that Black email aliases are 1.0 percentage point more likely to face follow-up screening questions when there is no signal (*p* = 0.006), which translates to a 58.4 percent increase. However, when there is a signal the effect of race is statistically insignificant (*p* = 0.685). Next, Black email aliases were 4.6 percentage points less likely to be told that anyone can apply when there is no signal (*p* < 0.000), which translates to a 20.7 percent decrease. In the signal condition the effect of race is statistically insignificant (*p* = 0.114). Once again, the treatment effects of race are significantly different across signal conditions for the likelihood of follow-up screening questions (*p* = 0.012) and for the likelihood of being told that anyone can apply (*p* = 0.003). When we test the robustness of this result in a multinomial logistic regression (see SI Appendix S3.8), we find that Black email aliases are more likely to be told that "Not everyone can apply" and less likely to be told "Yes, anyone can apply" when there is no signal, with no response as the baseline.

Finally, when we examine our proxy measures for psychological costs we find that Black email aliases were 7.6 percentage points (*p* < 0.000) and 3.9 percentage points (*p* < 0.000) less likely to receive an email with a greeting or salutation, respectively, when there was no signal of bureaucratic success criteria. These treatment effects translate to a 18.1 percent decrease in the likelihood of getting a greeting and a 13.3 percent decrease in the likelihood of getting a salutation for Black email aliases in the no signal condition. However, with the introduction of the signal, the effect of race becomes statistically insignificant for the inclusion of a greeting (*p* = 0.085) and salutation (*p* = 0.406). The treatment effect estimates for race are statistically different across signal conditions for the inclusion of a greeting (*p* = 0.008), but not for the inclusion of a salutation (*p* = 0.154).

We test the sensitivity of the results in multiple robustness checks available in SI Appendix Sect. 3. In SI Appendix section S3.1, we test whether the results are consistent across the first and second round of email inquiries. The results demonstrate the same dynamics across rounds, with a larger magnitude of effect sizes in the second round of emails, which could be due to an increase in workload in May as compared to March or the scarcity of open enrollment slots. In SI Appendix section S3.2, we test whether the results hold when we limit the analytical sample to those principals who responded within 4 weeks; The results are largely consistent but suffer from reduced statistical power and precision due to the smaller sample size. Next, we find that the results are robust when we drop all data from one of the twenty email accounts because it malfunctioned in round two (see SI Appendix S3.3); in the main results, we keep the data for this email account in round one but exclude all data from round two when the email account malfunctioned.

We also test alternative specifications in SI Appendix S3.4-S3.6 which all mirror the main results we present in Fig. 1. In SI Appendix S3.4 we show the results are robust after controlling for school demographic characteristics. In SI Appendix S3.5 we demonstrate that the results hold when we add email round fixed effects. In SI Appendix S3.6 we show the most conservative estimation approach, where we compare individual principals to themselves by including school fixed effects. Though some of the interaction coefficients between the signal and race conditions are no longer statistically significant, Black email aliases are consistently experiencing higher levels of administrative burden across cost categories when there is no signal of bureaucratic success criteria, in line with the main results. In SI Appendix S3.7 we test the efficacy of our randomization by implementing a standard OLS regression with our treatment dimensions as the dependent variable and a set of observable characteristics for each school as the independent variables. The results from this balance check show that none of the school characteristics are significantly predictive of the treatment assignment, providing support for the efficacy of randomization.

In SI Appendix S8.1 and S8.2, we test whether the results diverge based on whether the Black and White names were associated with higher than average or below average socioeconomic status. In SI Appendix Table S8.1, we provide data on the education level of each name, which is a commonly used proxy measure for socioeconomic status^35^. There is some variation in education level but the differences are small in magnitude, because we strategically selected names with similar education levels to avoid conflating race and socioeconomic status^36^. In the subgroup analysis in SI Appendix Table S8.2, we find that the results appear to be strongest for names that are associated with below average socioeconomic status, which provides important context for the interpretation of the results. We expand on this point below.

We also find that the results are robust to the exclusion of fixed effects in SI Appendix S9.1. Finally, we investigate whether the results are sensitive to the measurement of our outcome “Can Anyone Apply” by re-running the results with the outcome coded as a dichotomous indicator of whether the principal responded with any answer to the question rather than whether they answered “Yes, Anyone Can Apply”. This is important because charter schools are heterogeneous in terms of mission and the type of students they aim to serve, which could affect their response to our question of “Can Anyone Apply”; rather than providing exclusionary criteria, they could instead be clarifying the types of students they serve so parents do not waste time on the application process when their student will not be deemed eligible. In SI Appendix S10.1, we show that our results are robust to the alternative measurement of the dependent variable for “Can Anyone Apply”. Thus, the differential responsiveness across race that we observe is consistent regardless of measurement. It is possible, however, that the responses could include inaccurate information, but this is beyond the scope of our analysis and we encourage future research to take up this question.

## Discussion

In this study, we reported results from a nationwide email correspondence audit experiment including all charter school principals in the U.S. Our results show that charter principals engage in bureaucratic discrimination; Black email aliases are less likely to receive a response, are less likely to receive information on how to apply, are more likely to be asked follow-up questions about additional screening criteria, are less likely being told that everyone can apply, and are less likely to be greeted and offered a salutation in email correspondence. This suggests that charter school principals increase the learning, compliance and psychological costs that Black parents encounter when seeking information on how to enroll their children in charter schools. However, when direct information about a key bureaucratic success criteria is experimentally added to information requests–namely the child’s test scores and grades–these racial disparities are reduced significantly across all cost categories. These findings are present across different forms of charter schools and state charter regimes, and therefore seem to generalize broadly across the heterogeneous field of charter schools (see SI Appendix S7). Important heterogeneity did emerge, however, when we examined the intersection of race and socioeconomic status in the names we used in the study; our results are most pronounced for names that have lower than average socioeconomic status, which we measure using data on education level. This finding suggests that higher socioeconomic status may override the performance signal, but we do encourage caution in the interpretation of these results. Ultimately, the differences in socioeconomic status are extremely small in magnitude across our names because we balanced the socioeconomic status of the Black and White names in our selection process. Moving forward, we encourage future audit studies to investigate the intersection of race and socioeconomic status explicitly in the experimental design so that we have further evidence on the role of intersectionality in bureaucratic discrimination. Together, our findings are important because they uncover a causal mechanism of bureaucratic discrimination in access to public services that are delivered within competitive settings–a proposed causal mechanism that has not been systematically documented in previous research.

The main theoretical implication of our study is that bureaucratic discrimination is, in part, the result of the economic incentive structure of the organization delivering these services. In the charter school sector, principals use race as a shorthand to determine whether a client is desirable in terms of their organization’s bureaucratic success criteria. This implies that they engage in bureaucratic discrimination as a means of cream skimming. However, this ought not to mean that other motives for discrimination such as taste-based motives, or implicit biases and stereotypes do not play a role. Rather, in the context of public service delivery within marketized settings, bureaucratic discrimination as a means of cream skimming explains a significant portion of racial disparities in access and outcomes.

While the reported results support our underlying theory of bureaucratic discrimination as a means of cream-skimming, we also note that there was an unexpected effect of the bureaucratic success criteria signal that should be tested further in future research (This is a phenomenon that has been found in previous studies as well^25^.) The experimentally altered signal itself did not lead to a reduction in administrative burdens across the board; instead, there was heterogeneity across race in how the signal was interpreted. For White email aliases, the signal resulted in a higher level of administrative burdens, perhaps because the assumed group performance for Whites was better than “good” at baseline. On the other hand, particularly for schools with lower levels of proficiency, the signal resulted in a significantly higher response rate for Black students, which is in the expected direction (see SI Appendix Table 3.9). Given the disparities in group level performance on standardized tests and grade point average across White and Black students^37^, it is not altogether surprising that the signal had differential messages across White and Black aliases. In other words, with these group-level disparities in test scores and grades across race in mind, bureaucrats could be updating their beliefs about individual level performance in different directions. Regardless of this unexpected finding, we do still find support for the notion that *any information* about bureaucratic success criteria regardless of valence changes the likelihood of discrimination against African-American parents.

Against this background, our study provides important insights into the study of bureaucratic discrimination. Particularly, our research design allows us to move beyond the mere documentation of discriminatory behavior in public service delivery and documents one of the key causal mechanisms driving bureaucratic discrimination in marketized settings. We term this mechanism as bureaucratic discrimination as a means of cream-skimming. Understanding the underlying mechanisms of racial disparities in public service delivery more generally allows policymakers to explicitly design laws and regulations to tackle the disproportionate burdens African-Americans face when interacting with the state and its institutions. Our study also has larger implications for the design of public service delivery more generally. Indeed, past decades have seen a shift away from state-led provision of public services to the development of markets where public and private organizations compete with each other for clients in public service delivery. While our study did not experimentally manipulate the marketized setting of public services, our findings imply that the economic incentive structure within public service delivery organizations has unintended feedback effects, including a greater propensity for prioritizing clientele that fulfill organizational goals and performance metrics while minimizing costs. Indeed, these unanticipated feedback effects are rarely discussed by policymakers and academics but deserve greater attention in future research, given our findings.

In sum, our research offers an important contribution to the academic literature and societal conversations about racial equity and the role of institutional design in delivering public services for all. We find evidence for a key mechanism of why bureaucratic discrimination can come into being, namely as a means of cream-skimming. Our work provides evidence that scholars and policymakers should pay greater attention to the economic incentive structures within public service organizations and how they affect racial equity.

## Materials and methods

**Figure 2:**
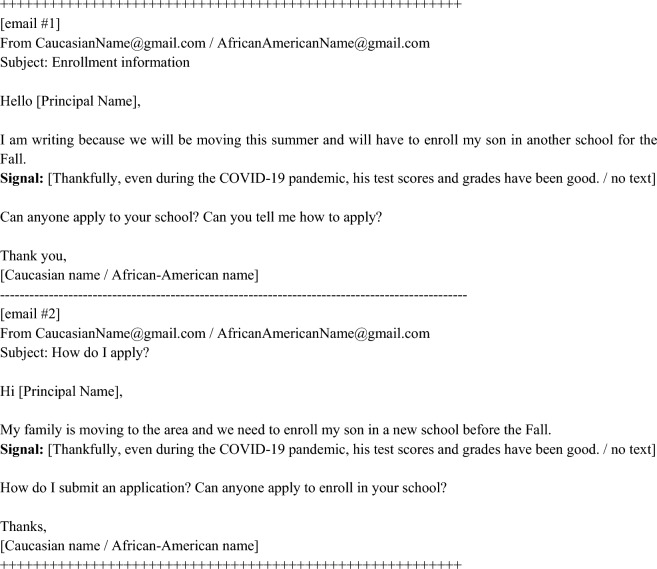
Randomly assigned email text. We pre-tested the emails in interviews with charter school principals to ensure the emails would appear like a real request that they might get from a parent. There are two email versions included to accommodate our cross-over experimental design whereby each principal received two emails total separated by a one-month washout period. We randomly assigned whether each principal received email number one or two in the first round and made sure they received the other version in round two.

The results reported here are from an email correspondence audit experiment approved by the Miami University Institutional Review Board (IRB); all methods were carried out in accordance with relevant guidelines and regulations, including the Miami University IRB approved waiver of informed consent. We sent out information requests to a comprehensive list of all charter school principals in the U.S., which we gathered by hand (see SI Appendix section S1.3). Using the ‘randomize’ package within Stata 16, randomization into experimental conditions was implemented with equal assignment probabilities using a reproducible seed. We experimentally vary the race of putative senders (factor #1) as well as bureaucratic success criteria of hypothetical students (factor #2) (see SI Appendix section S1.1, as well as Fig. 2). The set-up of experimental factors makes for a 2-by-2 factorial design with 4 experimental conditions, which we describe in detail in SI Appendix S1.1. Crossing these experimental factors allows us to explore the mechanism of frontline discrimination by testing whether charter school principals engage in bureaucratic discrimination as a means of cream-skimming. Each school principal received two requests, separated by a one-month wash-out period first in March 2021 and then in May 2021. Since we sent two requests to each school principal, we cross-over experimental conditions, meaning that we sent out the opposite factors in round two (i.e., a Caucasian name in round #1 would become an African-American name in round #2). We block randomized by state and management organization type (EMO, CMO, or freestanding) so that we have balance across treatment conditions within state and management organization type.

For the first experimental factor, we signal the race of the putative sender by randomly assigning either a Caucasian or an African-American female name to be included in the email address and in the signature at the end of the email (see below for the full email request). We selected the names by examining socio-economic status (SES) connotations of the 20 most common female African-American and Caucasian names used in previous studies. We selected 5 racially distinctive names for names with low-SES connotations and 5 racially distinctive names with high-SES connotations, to avoid our results being driven by the particularities of a single name or by SES^38^. In total, we utilized 10 White female email accounts and 10 Black female email accounts, making sure to not use any names that were used in prior audit studies of charter school principals (see SI Appendix Table S1.1).

As a second factor, we experimentally varied the direct signal of a prospective student’s future costliness: low costliness versus no signal. In Fig. 2, we include the two versions of the email requests we sent out to charter school principals. The principals were randomly assigned to see one version in the first round and the opposite version in the second round.

Table 2 summarizes our pre-registered primary outcomes, which include (1) the response rate within four weeks of sending the email, (learning cost), (2) whether they provided information on how to apply (learning cost), and (3) whether they asked follow-up questions that suggested screening criteria (compliance costs). We also measured a number of secondary outcomes including whether the principal answered “yes anyone can apply” versus no or no answer (compliance cost) and whether the response included a greeting or salutation (psychological cost) (We classify the “can anyone apply” question as a compliance cost because if not everyone can apply, that implies that there is some verification process where they have to prove eligibility, which increases compliance costs.)

**Table 2:** Summary of Pre-registered Dependent Variables.

Dependent Variable	Measurement	Administrative Burden	Primary outcome	Secondary outcome
*Response*: Any active and direct response to our inquiry is counted as (1), otherwise (0)	Yes/no	Learning cost	X	
*How to Apply*: Information on how to apply for the school is provided (including posting a link)?	Yes/no	Learning cost	X	
*Follow-up*: Asking follow-up about kid or parent that may suggest it's an application criteria	Yes/no	Compliance cost	X	
*Can anyone apply*: Do they say the school is open to anyone? Or do they suggest that there are some eligibility/verification criteria?	Yes vs. no/no response	Compliance cost		X
*Greeting*: Response involves a greeting	Yes/no	Psychological cost		X
*Salutation*: Response involves a salutation	Yes/no	Psychological cost		X

### Supplementary Information

Supplementary Information.

## Data Availability

All data are available in the supplementary materials.
