# Supplementary material for: Racial Discrimination and Administrative Burden in Access to Public Services
Source: Sci Rep. 2024 Jan 11;14:1071. doi: 10.1038/s41598-023-50936-1 (PMC10784545; doi:10.1038/s41598-023-50936-1)
Supplement: Supplementary file 1 — Supplementary Information. [file 41598_2023_50936_MOESM1_ESM.docx]

**Supporting Information for Racial Discrimination and Administrative Burden in Access to Public Services**

Elizabeth Bell Sebastian Jilke

Corresponding author: Elizabeth Bell

Email: elizabethbell012@gmail.com

**Contents**

S1 Audit methodology and experimental design

S1.1 Experimental design

S1.2 Data description and contact list

S1.3 Measurement of dependent variables

S2 Main Results

S2.1 Subgroup Regression Results for Racial Discrimination, by Signal Condition

S2.2 Interaction Regression Results

S2.3 Subgroup Regression Results for Racial Discrimination, by Signal Condition with Block Randomization Fixed Effects

S3 Supplementary Analysis

S3.1 Average treatment effects, by round

S3.2 Average treatment effects, without conditioning on response

S3.3 Average treatment effects, when dropping one email account

S3.4 Average treatment effects, when school covariates are included

S3.5 Average treatment effects, with Round fixed effects

S3.6 Average treatment effects, with school fixed effects

S3.7 Balance check

S3.8 Multinomial Logistic Regression Results for "Can Anyone Apply"

S4 AEA Pre-registration

S5 Example email responses and coding

S6 Pre-Study Conjoint Experiment Results

S7 Heterogenous Treatment Effects

S7.1 Subgroup Analysis, by State Pro-Charter Policy Score

S7.2 Subgroup Analysis, by Management Organization Type

S8 Socioeconomic Status of Racialized Names

S8.1 Descriptive Statistics of Each Racialized Name

S8.2 Subgroup Analysis, by Socioeconomic Status of Black and White Names

S9 Results without Fixed Effects

S9.1 Average treatment effects, without fixed effects

**S1 Audit methodology and experimental design**

**S1.1 Experimental design**

We sent information requests to charter school principals via email, experimentally varying the names of email senders. The email text was pre-tested in an interview with a charter school principal prior to the launch of the study to ensure that the email looked like a real request from a parent and sent during a time period where many parents are attempting to enroll their students in charter schools. In total, we utilized 10 White female email accounts and 10 Black female email accounts, making sure to not use any names that were used in prior audit studies of charter school principals, which we present in Table S1.1 below.

**Table S1.1 Validated African-American and Caucasian names**

|  | **Black Name** | **Caucasian Name** |
| --- | --- | --- |
| 1 | Alexus Banks | Abigail Smith* |
| 2 | Deja Mosley | Amy Mueller |
| 3 | Ebony Washington | Caitlin Schneider |
| 4 | Jada Moseley | Claire Schwartz |
| 5 | Jazmine Jefferson | Emily Schmidt |
| 6 | Keisha Rivers | Holly Schroeder |
| 7 | Kiara Jackson | Katelyn Miller* |
| 8 | Latoya Rivers | Katie Novak |
| 9 | Precious Washington | Madeline Haas |
| 10 | Shanice Booker | Molly Kruger |

* indicates slight abbreviation from CENSUS spelling

As a second factor, we experimentally varied the direct signal of a prospective student’s future costliness: low costliness versus no signal. We do not include a signal of high costliness (bad grades and behavior) because we are interested in the presence of a direct signal that replaces the need to statistically discriminate. In addition, it is reasonable to expect that a negative signal (bad grades and behavior) is differentially perceived for white and black students, making it a difficult to interpret it as a comparison base to the ‘no signal’ condition.

**S1.2 Measurement of dependent variables**

For our dependent variables, we capture elements of the principals’ reply that can be objectively measured. We do not include dependent variables that may have positive or negative theoretical valence; for instance, we do not include whether the principal offered to meet with the parent because they could either be an indication that the principal is trying to increase compliance costs (by requiring an in-person meeting) or reduce psychological costs (by extending an invitation for a meeting that would reduce stress and anxiety by offering personalized support).

**S1.3 Data description and contact list**

We provide a deidentified version of our data in a repository on the Open Science Framework DOI 10.17605/OSF.IO/XM9AG that can be accessed [here](https://osf.io/xm9ag/?view_only=ca8f95e096f44579a4055f330da08e0f). To construct the contact list, we identified the full list of charter schools operating in the U.S. through the National Center for Education Statistics (NCES) and dropped 82 schools identified as closed down or “not operational”. We also dropped all schools designated as “alternative schools” (i.e., juvenile justice schools) and virtual/cyber schools (546 schools total). Next, we contacted state agency officials and conducted online searches to record the email addresses for the principal or head of school. In the process of creating the final set of schools, we dropped schools where the principal emails were not accessible online (238 schools) and where the same principal was serving in multiple schools (~400 schools). This produced a dataset with a single principal contact within each of 5,850 schools.

To construct our analytical dataset, we leverage data from multiple sources including the National Alliance for Public Charter Schools (NAPCS) on proficiency rates and state pro-charter policy scores, the National Center for Education Statistics (NCES) on school demographic characteristics, and our hand-coded dependent variables capturing the responses from charter school principals. In constructing our analytical sample, we also dropped all principals who we failed to contact (495 schools) and we dropped observations for one email account that encountered an error in sending out emails (273 observations). This email account had a disproportionate number of failed contacts and after investigating further, we found that google blocked the emails that we attempted to send.

In the coding process, we began by having two research assistants code a sample of the data (20 emails) based on an initial codebook including our pre-registered set of dependent variables. Then, we checked intercoder reliability scores and reconciled differences in interpretation of the codebook and added exploratory outcomes based on what emerged from the email data. We completed two separate rounds of “training codes” until we achieved at least 80% intercoder reliability on every one of the pre-registered primary outcomes.

In the table below, we describe the key independent and dependent variables in the confirmatory analysis.

| **Table S1.2** **Descriptive Statistics** |  |  |  |  |  |
| --- | --- | --- | --- | --- | --- |
| Variable | N | Mean | Std. Dev. | Min | Max |
| *School Characteristics* |  |  |  |  |  |
| American Indian or Alaska Native Students | 3,357 | 4.39 | 26.72 | 0 | 1334 |
| Asian Students | 3,593 | 21.11 | 60.50 | 0 | 1322 |
| Hispanic students | 4,589 | 163.24 | 232.89 | 0 | 2863 |
| Black students | 4,469 | 122.42 | 216.28 | 0 | 4369 |
| White Students | 4,522 | 158.97 | 306.60 | 0 | 7676 |
| Pacific Islander Students | 3,030 | 2.20 | 13.47 | 0 | 457 |
| Multi-Race Students | 4,397 | 18.79 | 34.94 | 0 | 761 |
| Total Enrollment | 4,637 | 477.98 | 513.58 | 0 | 11173 |
| Free and Reduced-Price Lunch enrollment | 3,308 | 276.33 | 319.17 | 0 | 5004 |
| Pupil-Teacher Ratio | 4,135 | 22.25 | 113.80 | -0.02 | 5207.69 |
| English Language Arts Proficiency | 5,085 | 0.59 | 0.49 | 0 | 1 |
| Math Proficiency | 5,085 | 0.58 | 0.49 | 0 | 1 |
| Number of Students take ELA exam | 4,266 | 250.92 | 288.57 | 1 | 6596 |
| Number of Students take Math exam | 4,270 | 249.92 | 297.30 | 1 | 7948 |
| State Pro-Charter Policy Score | 5,081 | 0.62 | 0.48 | 0 | 1 |
| Management Organization Type-CMO | 5,083 | 0.22 | 0.42 | 0 | 1 |
| Management Organization Type-EMO | 5,083 | 0.12 | 0.32 | 0 | 1 |
| Management Organization Type-Freestanding | 5,083 | 0.63 | 0.48 | 0 | 1 |
| *Dependent Variables* |  |  |  |  |  |
| Response | 5,085 | 0.45 | 0.50 | 0 | 1 |
| Answered How to Apply | 5,085 | 0.30 | 0.46 | 0 | 1 |
| Answered Yes Anyone Can Apply | 5,085 | 0.21 | 0.40 | 0 | 1 |
| Greeting Included | 5,080 | 0.38 | 0.48 | 0 | 1 |
| Salutation Included | 5,080 | 0.27 | 0.45 | 0 | 1 |
| Follow Up Questions Asked | 5,085 | 0.03 | 0.15 | 0 | 1 |
| *Note:* The number of observations is different from the results tables because we limit it to one observation per school. We also only report on schools in our analytical sample (those that we failed to contact are dropped). | | | | | |

**S2 Main Results Tables**

| **Table S2.1** Subgroup Regression Results for Racial Discrimination, by Signal Condition | | | | | | | |
| --- | --- | --- | --- | --- | --- | --- | --- |
|  | **Black** | **SE** | **P-Value** | **Constant** | **SE** | **N** | **R-squared** |
| **Primary Outcomes** |  |  |  |  |  |  |  |
| Response, No Signal | **-0.074***** | (0.008) | 0.000 | 0.484*** | (0.015) | 4,899 | 0.035 |
| Response, Signal | -0.024 | (0.014) | 0.080 | 0.413*** | (0.019) | 4,994 | 0.041 |
| Answered How to Apply, No Signal | **-0.066***** | (0.008) | 0.000 | 0.345*** | (0.013) | 4,899 | 0.027 |
| Answered How to Apply, Signal | -0.019 | (0.010) | 0.055 | 0.287*** | (0.013) | 4,994 | 0.026 |
| Follow Up Questions Asked, No Signal | **0.010**** | (0.004) | 0.006 | 0.018*** | (0.002) | 4,894 | 0.009 |
| Follow Up Questions Asked, Signal | 0.001 | (0.002) | 0.685 | 0.022*** | (0.002) | 4,986 | 0.018 |
| **Secondary Outcomes** |  |  |  |  |  |  |  |
| Answered Yes Anyone Can Apply, No Signal | -0.046*** | (0.007) | 0.000 | 0.222*** | (0.007) | 4,899 | 0.028 |
| Answered Yes Anyone Can Apply, Signal | -0.016 | (0.010) | 0.114 | 0.174*** | (0.008) | 4,994 | 0.026 |
| Greeting Included, Signal | -0.076*** | (0.010) | 0.000 | 0.421*** | (0.011) | 4,897 | 0.034 |
| Greeting Included, No Signal | -0.024 | (0.014) | 0.085 | 0.359*** | (0.013) | 4,987 | 0.040 |
| Salutation Included, Signal | -0.039*** | (0.010) | 0.000 | 0.296*** | (0.008) | 4,896 | 0.023 |
| Salutation Included, No Signal | -0.011 | (0.013) | 0.406 | 0.254*** | (0.009) | 4,987 | 0.029 |
| Note: Each model includes state fixed effects to increase precision. Bolded coefficients are significant after Benjamini Hochberg multiple comparison adjustment (we only include primary outcomes in the multiple comparison adjustment). Robust standard errors clustered by management organization and school identifier in parentheses *** p<0.001, ** p<0.01, * p<0.05 | | | | | | | |

| **Table S2.2.** Regression Results for Racial Discrimination Interacted with Signal Condition | | | | | | |  |
| --- | --- | --- | --- | --- | --- | --- | --- |
|  |  |  |  |  |  |  |  |
|  | Response | Answered How to Apply | Follow Up Questions Asked | Answered Yes Anyone Can Apply | Greeting Included | Salutation Included |  |
| **Black** | -0.074*** | -0.065*** | 0.011** | -0.045*** | -0.075*** | -0.039*** |  |
| SE | (0.008) | (0.007) | (0.004) | (0.007) | (0.010) | (0.009) |  |
| P-value | 0.000 | 0.000 | 0.007 | 0.000 | 0.000 | 0.000 |  |
| **Signal** | -0.072*** | -0.059*** | 0.004 | -0.048*** | -0.064*** | -0.043*** |  |
| SE | (0.011) | (0.008) | (0.003) | (0.008) | (0.009) | (0.007) |  |
| P-value | 0.000 | 0.000 | 0.219 | 0.000 | 0.000 | 0.000 |  |
| **Black*Signal** | 0.051** | 0.048*** | -0.009* | 0.031** | 0.053** | 0.028 |  |
| SE | (0.018) | (0.012) | (0.004) | (0.010) | (0.020) | (0.020) |  |
| P-value | 0.005 | 0.000 | 0.012 | 0.003 | 0.008 | 0.154 |  |
|  |  |  |  |  |  |  |  |
| Constant | 0.485*** | 0.345*** | 0.018*** | 0.222*** | 0.421*** | 0.296*** |  |
|  | (0.013) | (0.012) | (0.002) | (0.007) | (0.009) | (0.007) |  |
|  |  |  |  |  |  |  |  |
| Observations | 9,895 | 9,895 | 9,882 | 9,895 | 9,886 | 9,885 |  |
| R-squared | 0.037 | 0.025 | 0.009 | 0.024 | 0.035 | 0.025 |  |
| Note: Each model includes state fixed effects to increase precision. Robust standard errors clustered by management organization and school identifier in parentheses *** p<0.001, ** p<0.01, * p<0.05 | | | | | | |  |

| **Table S2.3.** Subgroup Regression Results for Racial Discrimination, by Signal Condition with Block Randomization Fixed Effects | | | | | | | |
| --- | --- | --- | --- | --- | --- | --- | --- |
|  | **Black** | **SE** | **P-Value** | **Constant** | **SE** | **N** | **R-squared** |
| **Primary Outcomes** |  |  |  |  |  |  |  |
| Response, No Signal | **-0.0733***** | (0.008) | 0.000 | 0.484*** | (0.006) | 4,882 | 0.061 |
| Response, Signal | -0.023 | (0.013) | 0.067 | 0.413*** | (0.008) | 4,976 | 0.067 |
| Answered How to Apply, No Signal | **-0.067***** | (0.008) | 0.000 | 0.346*** | (0.005) | 4,882 | 0.051 |
| Answered How to Apply, Signal | -0.018* | (0.009) | 0.038 | 0.286*** | (0.006) | 4,976 | 0.052 |
| Follow Up Questions Asked, No Signal | **0.011**** | (0.004) | 0.008 | 0.018*** | (0.003) | 4,882 | 0.037 |
| Follow Up Questions Asked, Signal | 0.001 | (0.002) | 0.707 | 0.022*** | (0.001) | 4,976 | 0.029 |
| **Secondary Outcomes** |  |  |  |  |  |  |  |
| Answered Yes Anyone Can Apply, No Signal | -0.046*** | (0.007) | 0.000 | 0.222*** | (0.005) | 4,882 | 0.050 |
| Answered Yes Anyone Can Apply, Signal | -0.015 | (0.009) | 0.092 | 0.174*** | (0.005) | 4,976 | 0.048 |
| Greeting Included, Signal | -0.076*** | (0.011) | 0.000 | 0.421*** | (0.006) | 4,880 | 0.055 |
| Greeting Included, No Signal | -0.024 | (0.014) | 0.074 | 0.359*** | (0.008) | 4,969 | 0.064 |
| Salutation Included, Signal | -0.039*** | (0.010) | 0.000 | 0.296*** | (0.006) | 4,879 | 0.047 |
| Salutation Included, No Signal | -0.010 | (0.013) | 0.431 | 0.253*** | (0.006) | 4,969 | 0.049 |
| Note: Each model includes randomization block fixed effects (state + management organization type) to increase precision. Bolded coefficients are significant after Benjamini Hochberg multiple comparison adjustment (we only include primary outcomes in the multiple comparison adjustment). Robust standard errors clustered by management organization and school identifier in parentheses *** p<0.001, ** p<0.01, * p<0.05 | | | | | | | |

**S3 Supplementary Analysis**

**Table S3.1 Average treatment effects, by Round**

|  | **Round 1** | | | | | | | | | | | |
| --- | --- | --- | --- | --- | --- | --- | --- | --- | --- | --- | --- | --- |
|  | Response | Response | Answered How to Apply | Answered How to Apply | Follow Up Questions Asked | Follow Up Questions Asked | Answered Yes Anyone Can Apply | Answered Yes Anyone Can Apply | Greeting Included | Greeting Included | Salutation Included | Salutation Included |
|  | **No Signal** | **Signal** | **No Signal** | **Signal** | **No Signal** | **Signal** | **No Signal** | **Signal** | **No Signal** | **Signal** | **No Signal** | **Signal** |
| Black | -0.0406* | -0.0313* | -0.0390*** | -0.0215 | 0.0118* | -0.003 | -0.0243** | -0.0203 | -0.0525* | -0.0249 | -0.025 | -0.0113 |
| SE | (0.019) | (0.014) | (0.013) | (0.012) | (0.005) | (0.003) | (0.008) | (0.013) | (0.022) | (0.013) | (0.019) | (0.018) |
| P-value | 0.0352 | 0.0303 | 0.0034 | 0.0662 | 0.0187 | 0.2200 | 0.0024 | 0.1180 | 0.0190 | 0.0593 | 0.1970 | 0.5340 |
| Constant | 0.503*** | 0.426*** | 0.344*** | 0.279*** | 0.0224*** | 0.0244*** | 0.240*** | 0.193*** | 0.434*** | 0.354*** | 0.313*** | 0.253*** |
|  | (0.022) | (0.025) | (0.016) | (0.018) | (0.003) | (0.002) | (0.007) | (0.012) | (0.017) | (0.015) | (0.010) | (0.012) |
|  |  |  |  |  |  |  |  |  |  |  |  |  |
| Observations | 2,544 | 2,536 | 2,544 | 2,536 | 2,544 | 2,536 | 2,544 | 2,536 | 2,542 | 2,533 | 2,542 | 2,533 |
| R-squared | 0.046 | 0.041 | 0.034 | 0.027 | 0.020 | 0.026 | 0.034 | 0.035 | 0.045 | 0.041 | 0.030 | 0.040 |
|  | **Round 2** | | | | | | | | | | | |
|  | Response | Response | Answered How to Apply | Answered How to Apply | Follow Up Questions Asked | Follow Up Questions Asked | Answered Yes Anyone Can Apply | Answered Yes Anyone Can Apply | Greeting Included | Greeting Included | Salutation Included | Salutation Included |
|  | **No Signal** | **Signal** | **No Signal** | **Signal** | **No Signal** | **Signal** | **No Signal** | **Signal** | **No Signal** | **Signal** | **No Signal** | **Signal** |
| Black | -0.119*** | -0.0145 | -0.0999*** | -0.0166 | 0.009* | 0.0054 | -0.0749*** | -0.00964 | -0.108*** | -0.0223 | -0.058*** | -0.0087 |
| SE | (0.016) | (0.017) | (0.012) | (0.013) | (0.004) | (0.003) | (0.011) | (0.011) | (0.014) | (0.019) | (0.012) | (0.014) |
| P-value | 0.0000 | 0.4060 | 0.0000 | 0.2100 | 0.0408 | 0.1100 | 0.0000 | 0.3660 | 0.0000 | 0.2440 | 0.0000 | 0.5400 |
| Constant | 0.467*** | 0.400*** | 0.348*** | 0.296*** | 0.0127*** | 0.0197*** | 0.205*** | 0.155*** | 0.409*** | 0.364*** | 0.278*** | 0.255*** |
|  | (0.012) | (0.015) | (0.011) | (0.011) | (0.002) | (0.002) | (0.009) | (0.007) | (0.013) | (0.012) | (0.012) | (0.009) |
|  |  |  |  |  |  |  |  |  |  |  |  |  |
| Observations | 2,352 | 2,456 | 2,352 | 2,456 | 2,352 | 2,456 | 2,352 | 2,456 | 2,352 | 2,452 | 2,351 | 2,452 |
| R-squared | 0.044 | 0.052 | 0.041 | 0.042 | 0.011 | 0.022 | 0.042 | 0.038 | 0.041 | 0.052 | 0.034 | 0.030 |
| *Note:* Each model includes state fixed effects to increase precision. Robust standard errors clustered by management organization and school identifier in parentheses *** p<0.001, ** p<0.001, * p<0.05 | | | | | | | | | | | | |

**Table S3.2 Average treatment effects, without conditioning on response**

|  |  |  |  |  |  |  |  |  |  |  |  |
| --- | --- | --- | --- | --- | --- | --- | --- | --- | --- | --- | --- |
|  | Answered How to Apply | Answered How to Apply | Follow Up Questions Asked | Follow Up Questions Asked | Answered Yes Anyone Can Apply | Answered Yes Anyone Can Apply | Greeting Included | Greeting Included | Salutation Included | Salutation Included |  |
|  | **No Signal** | **Signal** | **No Signal** | **Signal** | **No Signal** | **Signal** | **No Signal** | **Signal** | **No Signal** | **Signal** |  |
| Black | -0.041*** | -0.0026 | 0.0325*** | 0.00588 | -0.031 | -0.012 | -0.0238* | -0.00538 | 0.0173 | 0.0133 |  |
| SE | (0.011) | (0.011) | (0.007) | (0.005) | (0.017) | (0.020) | (0.012) | (0.013) | (0.018) | (0.019) |  |
| P-value | 0.0004 | 0.8060 | 0.0000 | 0.1990 | 0.0622 | 0.5540 | 0.0424 | 0.6770 | 0.3440 | 0.4790 |  |
| Constant | 0.712*** | 0.692*** | 0.0366*** | 0.0535*** | 0.458*** | 0.420*** | 0.868*** | 0.868*** | 0.610*** | 0.614*** |  |
|  | (0.009) | (0.007) | (0.004) | (0.003) | (0.009) | (0.009) | (0.009) | (0.013) | (0.009) | (0.013) |  |
|  |  |  |  |  |  |  |  |  |  |  |  |
| Observations | 2,192 | 1,999 | 2,194 | 2,005 | 2,194 | 2,005 | 2,192 | 1,998 | 2,191 | 1,998 |  |
| R-squared | 0.033 | 0.029 | 0.023 | 0.028 | 0.027 | 0.034 | 0.056 | 0.046 | 0.024 | 0.026 |  |
| Note: This model only includes the principals who responded to test the robustness of the results for all of the outcomes, except for response rate. Each model includes state fixed effects to increase precision. Robust standard errors clustered by management organization and school identifier in parentheses *** p<0.001, ** p<0.01, * p<0.05 | | | | | | | | | | | |

**Table S3.3 Average treatment effects, when we drop one email account due to email account error**

|  | Response | Response | Answered How to Apply | Answered How to Apply | Follow Up Questions Asked | Follow Up Questions Asked | Answered Yes Anyone Can Apply | Answered Yes Anyone Can Apply | Greeting Included | Greeting Included | Salutation Included | Salutation Included |
| --- | --- | --- | --- | --- | --- | --- | --- | --- | --- | --- | --- | --- |
|  | **No Signal** | **Signal** | **No Signal** | **Signal** | **No Signal** | **Signal** | **No Signal** | **Signal** | **No Signal** | **Signal** | **No Signal** | **Signal** |
| Black | -0.068*** | -0.0123 | -0.059*** | -0.0107 | 0.0101* | 0.00127 | -0.042*** | -0.0129 | -0.071*** | -0.0121 | -0.036*** | -0.0019 |
| SE | (0.008) | (0.013) | (0.008) | (0.010) | (0.004) | (0.002) | (0.007) | (0.009) | (0.010) | (0.014) | (0.009) | (0.014) |
| P-value | 0.0000 | 0.3590 | 0.0000 | 0.2630 | 0.0168 | 0.5830 | 0.0000 | 0.1750 | 0.0000 | 0.3820 | 0.0002 | 0.8920 |
| Constant | 0.484*** | 0.414*** | 0.345*** | 0.287*** | 0.0178*** | 0.0222*** | 0.222*** | 0.174*** | 0.421*** | 0.359*** | 0.296*** | 0.254*** |
|  | (0.015) | (0.019) | (0.013) | (0.013) | (0.002) | (0.002) | (0.007) | (0.008) | (0.010) | (0.013) | (0.008) | (0.009) |
|  |  |  |  |  |  |  |  |  |  |  |  |  |
| Observations | 4,782 | 4,866 | 4,782 | 4,866 | 4,782 | 4,866 | 4,782 | 4,866 | 4,780 | 4,859 | 4,779 | 4,859 |
| R-squared | 0.035 | 0.042 | 0.026 | 0.027 | 0.009 | 0.018 | 0.027 | 0.026 | 0.035 | 0.040 | 0.024 | 0.029 |
| Note: This model tests the robustness of the results when we exclude the email account in Round 1 that encountered a technical error in Round 2 (Alexus Banks). In the main results, the Alexus Banks email account is included in Round 1 but not Round 2 because of the email error. Each model includes state fixed effects to increase precision. Robust standard errors clustered by management organization and school identifier in parentheses *** p<0.001, ** p<0.01, * p<0.05 | | | | | | | | | | | | |

| **Table S3.4 Average treatment effects, with school covariate controls** | | | | | | | | | | | |  | |  | |
| --- | --- | --- | --- | --- | --- | --- | --- | --- | --- | --- | --- | --- | --- | --- | --- |
|  |  |  |  |  |  |  |  |  |  |  |  | |  | |  |
|  | Response | Response | Answered How to Apply | Answered How to Apply | Follow Up Questions Asked | Follow Up Questions Asked | Answered Yes, Anyone Can Apply | Answered Yes Anyone Can Apply | Greeting Included | Greeting Included | Salutation Included | | Salutation Included | |  |
|  | **No Signal** | **Signal** | **No Signal** | **Signal** | **No Signal** | **Signal** | **No Signal** | **Signal** | **No Signal** | **Signal** | **No Signal** | | **Signal** | |  |
| **Black** | -0.080*** | -0.0163 | -0.065*** | -0.0156 | 0.00849 | 0.00103 | -0.043*** | -0.0133 | -0.078*** | -0.0172* | -0.041*** | | -0.00342 | |  |
| SE | (0.009) | (0.010) | (0.009) | (0.009) | (0.005) | (0.002) | (0.007) | (0.007) | (0.011) | (0.007) | (0.012) | | (0.008) | |  |
| P-value | 0.0000 | 0.1140 | 0.0000 | 0.0758 | 0.0761 | 0.6510 | 0.0000 | 0.0649 | 0.0000 | 0.0224 | 0.0005 | | 0.6510 | |  |
| **Percent White Enrollment** | 0.102*** | 0.133*** | 0.0895*** | 0.144*** | 0.00968 | 0.0120** | 0.0795*** | 0.101*** | 0.0487** | 0.0823*** | 0.0550** | | 0.0847*** | |  |
| SE | (0.019) | (0.021) | (0.017) | (0.017) | (0.012) | (0.004) | (0.018) | (0.012) | (0.015) | (0.019) | (0.017) | | (0.016) | |  |
| P-value | 0.0000 | 0.0000 | 0.0000 | 0.0000 | 0.4250 | 0.0013 | 0.0000 | 0.0000 | 0.0017 | 0.0000 | 0.0015 | | 0.0000 | |  |
| **Total Enrollment** | -0.0161 | -0.0312* | -0.0247* | 0.00347 | -0.0169*** | -0.00632** | -0.0154* | -0.00511 | -0.00801 | -0.0186 | 0.00758 | | -0.00475 | |  |
| SE | (0.011) | (0.015) | (0.010) | (0.013) | (0.004) | (0.002) | (0.006) | (0.019) | (0.009) | (0.014) | (0.009) | | (0.014) | |  |
| P-value | 0.1410 | 0.0392 | 0.0140 | 0.7830 | 0.0001 | 0.0029 | 0.0115 | 0.7930 | 0.3930 | 0.1920 | 0.4210 | | 0.7260 | |  |
| **Teacher-to-Student Ratio** | -0.0233 | -0.0398 | -0.0245 | -0.034 | 0.00427 | 0.0128*** | 0.0113 | -0.036*** | -0.0194 | -0.0252 | -0.0149 | | -0.031* | |  |
| SE | (0.026) | (0.024) | (0.013) | (0.021) | (0.003) | (0.003) | (0.014) | (0.009) | (0.018) | (0.022) | (0.018) | | (0.014) | |  |
| P-value | 0.3670 | 0.0952 | 0.0605 | 0.1020 | 0.1950 | 0.0000 | 0.4250 | 0.0002 | 0.2900 | 0.2420 | 0.4140 | | 0.0273 | |  |
| Constant | 0.471*** | 0.402*** | 0.341*** | 0.253*** | 0.0219** | 0.0135*** | 0.193*** | 0.163*** | 0.419*** | 0.349*** | 0.280*** | | 0.241*** | |  |
|  | (0.015) | (0.015) | (0.013) | (0.012) | (0.007) | (0.002) | (0.010) | (0.010) | (0.011) | (0.013) | (0.013) | | (0.013) | |  |
|  |  |  |  |  |  |  |  |  |  |  |  | |  | |  |
| Observations | 4,354 | 4,433 | 4,354 | 4,433 | 4,354 | 4,433 | 4,354 | 4,433 | 4,352 | 4,427 | 4,351 | | 4,427 | |  |
| R-squared | 0.044 | 0.050 | 0.035 | 0.035 | 0.013 | 0.023 | 0.033 | 0.032 | 0.040 | 0.044 | 0.028 | | 0.034 | |  |
| Note: Each model includes state fixed effects to increase precision. Models include school demographic characteristics as control variables. Robust standard errors clustered by management organization and school identifier in parentheses *** p<0.001, ** p<0.01, * p<0.05 | | | | | | | | | | | | | | | |

**Table S3.5 Average treatment effects, with Round fixed effects**

|  |  |  |  |  |  |  |  |  |  |  |  |  |
| --- | --- | --- | --- | --- | --- | --- | --- | --- | --- | --- | --- | --- |
|  | Response | Response | Answered How to Apply | Answered How to Apply | Follow Up Questions Asked | Follow Up Questions Asked | Answered Yes Anyone Can Apply | Answered Yes Anyone Can Apply | Greeting Included | Greeting Included | Salutation Included | Salutation Included |
|  | **No Signal** | **Signal** | **No Signal** | **Signal** | **No Signal** | **Signal** | **No Signal** | **Signal** | **No Signal** | **Signal** | **No Signal** | **Signal** |
| Black | -0.076*** | -0.0236 | -0.067*** | -0.0188 | 0.0101** | 0.000872 | -0.047*** | -0.0154 | -0.077*** | -0.0237 | -0.041*** | -0.0111 |
| SE | (0.008) | (0.014) | (0.008) | (0.010) | (0.004) | (0.002) | (0.007) | (0.010) | (0.011) | (0.014) | (0.010) | (0.013) |
| P-value | 0.0000 | 0.0809 | 0.0000 | 0.0533 | 0.0062 | 0.6890 | 0.0000 | 0.1140 | 0.0000 | 0.0842 | 0.0001 | 0.4060 |
| Constant | 0.520*** | 0.422*** | 0.358*** | 0.277*** | 0.0235*** | 0.0227*** | 0.251*** | 0.191*** | 0.447*** | 0.353*** | 0.321*** | 0.252*** |
|  | (0.018) | (0.024) | (0.015) | (0.015) | (0.003) | (0.002) | (0.008) | (0.013) | (0.013) | (0.014) | (0.007) | (0.014) |
|  |  |  |  |  |  |  |  |  |  |  |  |  |
| Observations | 4,899 | 4,994 | 4,899 | 4,994 | 4,899 | 4,994 | 4,899 | 4,994 | 4,897 | 4,987 | 4,896 | 4,987 |
| R-squared | 0.040 | 0.041 | 0.028 | 0.027 | 0.011 | 0.018 | 0.033 | 0.028 | 0.037 | 0.040 | 0.027 | 0.029 |
| Note: This model tests the robustness of the results when we include round fixed effects in the model in addition to state fixed effects to increase precision. Robust standard errors clustered by management organization and school identifier in parentheses *** p<0.001, ** p<0.01, * p<0.05 | | | | | | | | | | | | |

**Table S3.6 Average treatment effects, with school fixed effects**

|  | Response | Answered How to Apply | Follow Up Questions Asked | Answered Yes Anyone Can Apply | Greeting Included | Greeting Included |
| --- | --- | --- | --- | --- | --- | --- |
| **Black** | -0.0395* | -0.0438* | 0.0166*** | -0.0235* | -0.0535* | -0.0234 |
| SE | (0.019) | (0.018) | (0.005) | (0.010) | (0.026) | (0.016) |
| P-value | 0.035 | 0.016 | 0.001 | 0.022 | 0.038 | 0.145 |
| **Signal** | -0.0489** | -0.0447** | 0.00844 | -0.0301* | -0.0484** | -0.0309** |
| SE | (0.016) | (0.016) | (0.005) | (0.014) | (0.016) | (0.011) |
| P-value | 0.002 | 0.006 | 0.065 | 0.036 | 0.002 | 0.007 |
| **Black*Signal** | -0.013 | 0.00598 | -0.0203*** | -0.012 | 0.0108 | -0.00135 |
| SE | (0.033) | (0.028) | (0.005) | (0.021) | (0.045) | (0.027) |
| P-value | 0.691 | 0.829 | 0.000 | 0.560 | 0.809 | 0.961 |
|  |  |  |  |  |  |  |
| Constant | 0.475*** | 0.341*** | 0.0156*** | 0.214*** | 0.417*** | 0.292*** |
|  | (0.010) | (0.010) | (0.003) | (0.008) | (0.010) | (0.007) |
|  |  |  |  |  |  |  |
| Observations | 9,657 | 9,657 | 9,657 | 9,657 | 9,645 | 9,644 |
| R-squared | 0.432 | 0.406 | 0.371 | 0.391 | 0.42 | 0.397 |
| *Note*: Each model includes school/principal fixed effects. We run the interaction model rather than the subgroup analysis which allows for us to estimate the effect of each treatment dimension within principals across the two rounds of email inquiries. Robust standard errors clustered by management organization in parentheses *** p<0.001, ** p<0.01, * p<0.05 | | | | | | |

**Table S3.7 Balance Check to Test Efficacy of Randomization**

|  | **Race (Black v. White)** | **Signal v. No Signal** |
| --- | --- | --- |
|  |  |  |
| FRL Enrollment | 0.0000 | 0.0000 |
| SE | (0.0001) | (0.0001) |
| P-value | 0.8040 | 0.5370 |
| American Indian/Alaska Native Enrollment | 0.0000 | 0.0000 |
| SE | (0.0008) | (0.0008) |
| P-value | 0.9780 | 0.9540 |
| Asian/Pacific Islander Enrollment | -0.0001 | 0.0000 |
| SE | (0.0005) | (0.0005) |
| P-value | 0.9110 | 0.9410 |
| Hispanic Enrollment | -0.0001 | 0.0000 |
| SE | (0.0005) | (0.0005) |
| P-value | 0.8910 | 0.9550 |
| Black Enrollment | -0.0001 | 0.0000 |
| SE | (0.0005) | (0.0005) |
| P-value | 0.9140 | 0.9490 |
| White Enrollment | 0.0000 | 0.0000 |
| SE | (0.0005) | (0.0005) |
| P-value | 0.9370 | 0.9850 |
| Multiple Races Enrollment | -0.0002 | -0.0001 |
| SE | (0.0007) | (0.0007) |
| P-value | 0.7980 | 0.8960 |
| Total Enrollment | 0.0000 | 0.0000 |
| SE | (0.0005) | (0.0005) |
| P-value | 0.9280 | 0.9840 |
| Pupil Teacher Ratio | 0.0000 | 0.0000 |
| SE | (0.0001) | (0.0001) |
| P-value | 0.9550 | 0.9270 |
| ELA Proficiency | 0.0027 | 0.0005 |
| SE | (0.0244) | (0.0244) |
| P-value | 0.9140 | 0.9840 |
| Math Proficiency | 0.0053 | -0.0116 |
| SE | (0.0244) | (0.0244) |
| P-value | 0.8290 | 0.6350 |
| ELA Testing Participation | 0.0000 | 0.0000 |
| SE | (0.0003) | (0.0003) |
| P-value | 0.9650 | 0.9970 |
| Math Testing Participation | 0.0000 | 0.0000 |
| SE | (0.0002) | (0.0002) |
| P-value | 1.0000 | 0.9940 |
| Constant | 0.481*** | 0.511*** |
|  | (0.0160) | (0.0160) |
|  | 0.0000 | 0.0000 |
|  |  |  |
| Observations | 3,722 | 3,722 |
| R-squared | 0.0000 | 0.0000 |
| Robust standard errors in parentheses *** p<0.001, ** p<0.01, * p<0.05; Limited to one observation per school and dropped schools with missing observable characteristics. | | |

**S3.8 Multinomial Logistic Regression Results for "Can Anyone Apply"**

In the table below, we provide a multinomial logistic regression analysis for our secondary outcome “can anyone apply”, in which we distinguish between responses that indicated: 1) everyone can not apply, 2) everyone can apply, and 3) no response.

| **Table S3.8 Multinomial Logistic Regression Results for "Can Anyone Apply"** | | | | |
| --- | --- | --- | --- | --- |
|  |  |  |  |  |
|  | **No Signal** | | **Signal** | |
|  | No | Yes | No | Yes |
|  | *(Baseline=No Response)* | | *(Baseline=No Response)* | |
| **Black** | 0.143** | -0.193** | 0.113 | -0.0312 |
| SE | (0.049) | (0.073) | (0.068) | (0.085) |
| P-value | 0.0033 | 0.0080 | 0.0943 | 0.7130 |
| Constant | 1.248*** | 0.902*** | 1.045*** | -0.679*** |
|  | (0.113) | (0.031) | (0.149) | (0.040) |
|  |  |  |  |  |
| Observations | 4,900 | 4,900 | 4,995 | 4,995 |
| Note: Each model includes state fixed effects to increase precision. Robust standard errors clustered by management organization and school identifier in parentheses *** p<0.001, ** p<0.01, * p<0.05 | | | | |

| **Table S3.9 Average treatment effects of signal across race, by proficiency** | | | | | | | |  |  |  |  |  |
| --- | --- | --- | --- | --- | --- | --- | --- | --- | --- | --- | --- | --- |
|  |  |  |  |  |  |  |  |  |  |  |  |  |
|  | Response | Response | Answered How to Apply | Answered How to Apply | Follow Up Questions Asked | Follow Up Questions Asked | Answered Yes Anyone Can Apply | Answered Yes Anyone Can Apply | Greeting Included | Greeting Included | Salutation Included | Salutation Included |
|  | **White** | **Black** | **White** | **Black** | **White** | **Black** | **White** | **Black** | **White** | **Black** | **White** | **Black** |
|  | *Below Average Math Proficiency* | | | | | | | | | | | |
| Signal | -0.082*** | 0.0346** | -0.057*** | 0.000111 | -0.00068 | 0.00123 | -0.0332** | 0.0129 | -0.064*** | 0.0423* | -0.0225 | 0.0187 |
| SE | (0.019) | (0.013) | (0.014) | (0.009) | (0.006) | (0.006) | (0.013) | (0.011) | (0.016) | (0.017) | (0.013) | (0.022) |
| P-value | 0.0000 | 0.0077 | 0.0000 | 0.9900 | 0.9020 | 0.8350 | 0.0090 | 0.2540 | 0.0001 | 0.0149 | 0.0916 | 0.3900 |
| Constant | 0.507*** | 0.290*** | 0.400*** | 0.300*** | 0.000433 | -0.00037 | 0.203*** | 0.0961*** | 0.313*** | 0.187*** | 0.196*** | 0.294*** |
|  | (0.012) | (0.004) | (0.009) | (0.003) | (0.004) | (0.002) | (0.008) | (0.003) | (0.010) | (0.005) | (0.008) | (0.007) |
|  |  |  |  |  |  |  |  |  |  |  |  |  |
| Observations | 2,098 | 2,001 | 2,098 | 2,001 | 2,098 | 2,001 | 2,098 | 2,001 | 2,096 | 1,999 | 2,095 | 1,999 |
| R-squared | 0.029 | 0.027 | 0.028 | 0.014 | 0.024 | 0.025 | 0.027 | 0.024 | 0.032 | 0.031 | 0.019 | 0.022 |
|  | *Above Average Math Proficiency* | | | | | | | | | | | |
| Signal | -0.062*** | -0.060*** | -0.058*** | -0.0186 | 0.00821* | -0.0101* | -0.058*** | -0.0384** | -0.061*** | -0.048** | -0.057*** | -0.0365* |
| SE | (0.012) | (0.017) | (0.010) | (0.013) | (0.004) | (0.004) | (0.010) | (0.015) | (0.011) | (0.016) | (0.012) | (0.018) |
| P-value | 0.0000 | 0.0005 | 0.0000 | 0.1530 | 0.0273 | 0.0119 | 0.0000 | 0.0090 | 0.0000 | 0.0036 | 0.0000 | 0.0454 |
| Constant | 0.555*** | 0.388*** | 0.494*** | 0.364*** | -0.00338* | 0.00596** | 0.318*** | 0.258*** | 0.496*** | 0.323*** | 0.318*** | 0.198*** |
|  | (0.046) | (0.050) | (0.042) | (0.054) | (0.002) | (0.002) | (0.027) | (0.030) | (0.043) | (0.039) | (0.030) | (0.021) |
|  |  |  |  |  |  |  |  |  |  |  |  |  |
| Observations | 2,957 | 2,839 | 2,957 | 2,839 | 2,957 | 2,839 | 2,957 | 2,839 | 2,955 | 2,836 | 2,955 | 2,836 |
| R-squared | 0.073 | 0.045 | 0.044 | 0.038 | 0.015 | 0.018 | 0.041 | 0.044 | 0.069 | 0.044 | 0.060 | 0.039 |
| Note: Each model includes state fixed effects to increase precision. Robust standard errors clustered by management organization and school identifier in parentheses *** p<0.001, ** p<0.01, * p<0.05 | | | | | | | | | | | | |

**S4 AEA Pre-registration**

**Link:** Bell, Elizabeth and Sebastian Jilke. 2021. "Racial Discrimination in Charter Schools: A Large-Scale Field Experiment Exploring the Causal Mechanism of Discrimination." AEA RCT Registry. May 03. <https://doi.org/10.1257/rct.7573-1.0>

**Project rationale**

Much of the literature that deals with cream-skimming/ cropping of charter schools is concerned with adverse student selection procedures. Arguably, there are different mechanisms through which charter schools can select prospective students. At the initial stage of the enrollment process, parents seek information on how to enlist their kid in school, including where and how to apply for a lottery if there exists one. At this stage, *administrative burdens* in accessing relevant information can be increased. Indeed, the availability of information is a crucial criterion for parents to learn about schools of choice and subsequently apply to them. But why would charter schools select certain types of students by increasing administrative burdens (i.e., learning and compliance costs)?

Charter schools are privately managed schools that compete for students with traditional public schools. More than public district schools, they are subject to competitive pressures to sustain on the educational marketplace. We predict that charters will prioritize students who they perceive easier-to-serve and therefore less costly. In addition, the No Child Left Behind Act performance regime induces incentives to focus on students that will meet bureaucratic success criteria like high standardized test scores. Charters are hence under double-pressure to sustain economically while at the same time they need to meet performance targets in terms of students’ academic achievements. It is therefore reasonable to expect that charter schools have developed an inherent incentive structure that leads them to focus on an easier-to-serve clientele.

*Direct signals* about the future performance and/or costliness of prospective students are often not available during initial information requests send to charter schools. This is the first entry point for unequal treatment. Drawing on the *statistical discrimination* framework, we argue that charter schools instead use *imperfect signals* of students’ future performance and costliness.

*H1: This means that in the absence of direct information, charter school principals will draw on population-based inferences about the average performance/ costliness of members of certain racial/ethnic groups, and use this population-specific statistical knowledge as a stereotype against individual applicants. As a consequence, racial/ethnic groups who are perceived to perform less well on average academically and hence potentially being costlier (like African-Americans and Latinx) will be discriminated against in accessing charter schools.*

*H2: We also predict that the likelihood of discrimination will depend on the management organization type, and specifically whether the charter is a non-profit, for-profit, or run by a public school district.*

To test these theoretical predictions, we will send out information requests to all charter school principals in the US in April and May of 2021. In the study, we will experimentally vary the race of putative senders as well as ‘cost criteria’ of hypothetical students. Crossing these experimental factors allows us to explore the mechanism of frontline discrimination by testing whether charter school principals engage in statistical discrimination as a means of cream-skimming. In addition, we expect heterogeneity in discrimination among charter schools based on their degree of market-orientation and overall educational mission. For instance, private for-profit schools that cater academically strong students will be more likely to discriminate against students they perceive as costly on the basis of test scores compared to nonprofit schools that focus on vulnerable students.

**Sample**

5,580 charter school principals (email-based)

**Intervention design**

We will send information requests to school principals via email, experimentally varying the names of email senders. Each school principal will receive two requests, separated by a one-month wash-out period. Names will signal a Caucasian versus an African-American female parent, and will be selected by examining socio-economic status connotations of the 20 most common female African-American and Caucasian names used in previous studies.[1] We aim to select 5 racially distinctive names which are perceived to be similar in terms of SES to avoid our results being driven by the particularities of a single name.

As a second factor, we will experimentally vary the direct signal of a prospective student’s future costliness: low costliness versus no signal. We do not include a signal of high costliness (bad grades and behavior) because we are interested in the presence of a direct signal that replaces the need to statistically discriminate. In addition, it is reasonable to expect that a negative signal (bad grades and behavior) is differentially perceived for white and black students, making it a difficult to interpret it as a comparison base to the ‘no signal’ condition. Lastly, we anticipate substantial heterogenous effects among different charter types and prefer to be well powered to detect these.

The set-up of experimental factors makes for a 2-by-2 factorial design with 4 experimental conditions. Since we will send two requests to each school principal, we will cross-over experimental conditions, meaning that we will send out the opposite factors in round two (i.e., a Caucasian name in round #1 would become an African-American name in round #1)

**Randomization Method**

Randomization done in STATA, blocked by state and management organization type (EMO, CMO, freestanding, or missing in a small set of cases)

**Randomization Unit**

Blocked by state and management organization, randomization is at the individual school level.

**Was the treatment clustered?**

Yes

**Sample size: planned number of clusters**

180 total clusters (45 states + 4 management organization types)

**Sample size: planned number of observations**

5,580 school principals, but they get two emails so 11,160 total

**Sample size (or number of clusters) by treatment arms**

For each of the four treatment groups (white/black*signal/no-signal) we will have 1,395 individual school principals that will be contacted twice, which will end up being 2,790 observations.

**INSTITUTIONAL REVIEW BOARDS (IRBs)**

**IRB Name**

Miami University

**IRB Approval Date**

2021-02-08

**IRB Approval Number**

01978r

**S5 Example Email Responses**

| **Email Text** | **Coding** |
| --- | --- |
| Good Afternoon, Thank you for your interest in registering your child at [School] for the 2021-2022 school year. To enroll your child at [School] we will need the following documents: enrollment application, a copy of the child’s birth certificate, a current report card, 2 proof of residence (water, light, or cable bill), your picture ID, and shots/physical record. The registration form is on our school website [WEBSITE]. If you are unable to upload the documents on the website, you can email the documents to [EMAIL].com or you can take them to the office. Our office hours are Mon-Fri from 9am-3:00pm. If you have any questions, please contact me at [PHONE NUMBER]. Thank you | Low learning costs—answered how to apply and responded  High compliance costs—asked for documentation and did not make it clear whether anyone can apply  Low psychological costs—provided a greeting and salutation |
| Molly,  Thank you for your interest in [School]! We are proud that our mission: that all students are safe, loved, and learning as they achieve excellence in academics and character has served our area so very well for the last twenty years. There are multiple ways to enroll, and we are open to all as a [STATE] public school academy. The easiest way for us to begin getting to know you and ensure a smooth process for you is for me to put you in touch with [NAME], who is included on this email and coordinates all of our enrollments. [NAME] will reach out to you to set up a personal tour when it is convenient for you as well as getting the paperwork process started. Again, thank you so much for expressing interest in joining our family!  Peace, | Low learning costs—answered how to apply and responded  Low compliance costs—no follow up questions and makes it clear anyone can apply  Low psychological costs—provided a greeting and salutation |
| Good morning Ms. Washington,  Thank you for inquiring about our program. Please complete the enrollment application and instructional form if interested in enrolling. When you come into the office, please bring the following required documents:  - Birth Certificate – (students)  - Current Immunization from NC - Health Record - Physical Exam if you are a new student to [CITY]  Please call the [County] Health Department - First-time appointments should call [PHONE NUMBER]  - Picture identification from parents/guardian (if student is under 18 years of age) and/or student ID  - Verification of Academic History  · Transcript  · Withdrawal Form  · Last report card or historical grades  - Verification of Special education information (if applicable)  · Current IEP  · Current 504 plan  - Health insurance card – private or Medicaid  - Proof of residence: copy of lease; property tax bill; utility bill (most recent gas, electric, water telephone or cable bill for the month of April 2021)  - Verification of Guardianship, if applicable  · Court Custody documents  · Department of Children Services Placement Letter  · Educational Guardianship documents  Thank you.  [PRINCIPAL NAME] | Low learning costs—answered how to apply and responded  High compliance costs—asked for documentation and did not make it clear whether anyone can apply  Low psychological costs—provided a greeting and salutation |
| Good morning and blessings Ebony Washington, Thank you for the information regarding enrollment with [School]. I have copied our Registrar, Ms. [NAME], who will be more than happy to respond or take any additional questions.  Anyone may to apply, and we will accept students as seats become available. Please apply at [SCHOOL]. We look forward to hearing from you, please feel free to give us a call if you have question. Have a blessed day! | Low learning costs—answered how to apply and responded  Low compliance costs— no follow up questions and makes it clear anyone can apply  Low psychological costs—provided a greeting and salutation |

**S6 Pre-Study Conjoint Experiment Results**

We sent out a conjoint experiment to all charter school principals in the US in April 2019. We sent invitations to participate in our survey to a comprehensive list of all charter school principals currently serving in the U.S., which we gathered by hand. To construct the contact list, we identified the full list of charter schools operating in the U.S. through the National Center for Education Statistics (NCES) and dropped 82 schools identified as closed down or “not operational”. Next, we contacted state agency officials and conducted online searches to record the email addresses for the principal or head of school. We collected many of the email addresses for the principals by emailing each of the state agencies to see if there was an up-to-date state directory. For the states with no state directory of charter school principals, we gathered this information by hand through searching the websites of each school for the principal’s contact information. For schools that did not list the principal’s email on the website, we utilized information from greatschools.com to recover additional email addresses. In the process of creating the final set of schools, we dropped schools where the principal emails were not accessible online (238 schools) and where the same principal was serving in multiple schools (~400 schools). In the cases where a single principal served at multiple schools, we randomly select the school that is kept in the dataset. This produced a dataset with a single principal contact within each of 6,396 schools.

The conjoint design asked principals to prioritize one of two hypothetical profiles of prospective students. Student profiles included randomly presented attributes, including gender, race/ethnicity, math and reading scores, English language learner status, special education needs, and parents’ occupation. By randomly presenting different levels of these attributes (including no information), we estimate the *average marginal component effect* of each student attribute separately and in combination with all others. In Table S6.1 and Figure S6.1 below, we present the results for this analysis based on the 490 respondents we received. When compared to the population of charter schools, our sample differs in some important ways. Our survey sample includes schools that have higher proportions of white students and lower proportions of students of color. Our survey sample also includes more magnet schools and schools with less impoverished student populations. Finally, our respondents reflect a higher proportion of rural schools and a lower proportion of urban schools that the population of charters across the country.


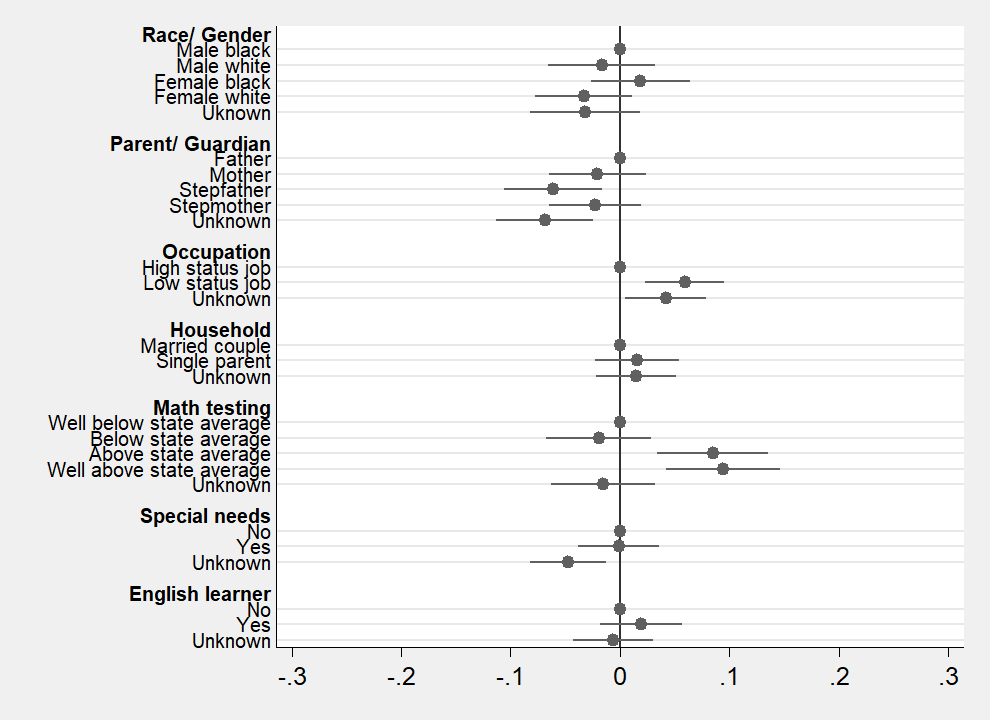


**Figure S6.1.** Treatment Effects for Each Student Characteristic on Likelihood of Prioritization by Charter School Principal

This figure presents the treatment effects of each student attribute on the likelihood that they were selected to be prioritized by charter school principals in our average marginal component effect analysis. (95% confidence intervals with standard errors clustered by principal; n=4660 nested in 490 respondents).

**Table S6.1**. Conjoint Results

| **Race/Gender** (Ref Male Black) | |
| --- | --- |
| Male White | -0.0165 |
|  | (0.0248) |
| Female Black | 0.0188 |
|  | (0.0231) |
| Female White | -0.0330 |
|  | (0.0226) |
| Unknown | -0.0321 |
|  | (0.0256) |
| **Parent/Guardian** (Ref Father) | |
| Mother | -0.0205 |
|  | (0.0225) |
| Stepfather | -0.0612*** |
|  | (0.0228) |
| Stepmother | -0.0225 |
|  | (0.0215) |
| Unknown | -0.0688*** |
|  | (0.0226) |
| **Occupation** (Ref High Status Job) | |
| Low Status Job | 0.0591*** |
|  | (0.0183) |
| Unknown | 0.0417** |
|  | (0.0189) |
| **Household Type** (Ref Married Couple) | |
| Single Parent | 0.0156 |
|  | (0.0194) |
| Unknown | 0.0150 |
|  | (0.0186) |
| **Math Test Scores** (Well below state average) | |
| Below state average | -0.0194 |
|  | (0.0243) |
| Above state average | 0.0847*** |
|  | (0.0259) |
| Well above state average | 0.0945*** |
|  | (0.0264) |
| Unknown | -0.0153 |
|  | (0.0241) |
| **Special Needs** (Ref No) |  |
| Yes | -0.00120 |
|  | (0.0187) |
| Unknown | -0.0475*** |
|  | (0.0176) |
| **English Language Learner** (Ref No) | |
| Yes | 0.0196 |
|  | (0.0191) |
| Unknown | -0.00637 |
|  | (0.0188) |
| Constant | 0.485*** |
|  | (0.0345) |
| Observations | 4,666 |
| R-squared | 0.020 |

Robust standard errors in parentheses clustered by principal *** p<0.01, ** p<0.05, * p<0.1

**S7 Heterogenous Treatment Effects**

**Table S7.1** Subgroup Analysis, by State Pro-Charter Policy Score

|  | Response | Response | Answered How to Apply | Answered How to Apply | Follow Up Questions Asked | Follow Up Questions Asked | Answered Yes Anyone Can Apply | Answered Yes Anyone Can Apply | Greeting Included | Greeting Included | Salutation Included | Salutation Included |
| --- | --- | --- | --- | --- | --- | --- | --- | --- | --- | --- | --- | --- |
|  | *Above Median Pro-Charter State Policy Score* | | | | | | | | | | | |
|  | **No Signal** | **Signal** | **No Signal** | **Signal** | **No Signal** | **Signal** | **No Signal** | **Signal** | **No Signal** | **Signal** | **No Signal** | **Signal** |
| Black | -0.086*** | -0.0166 | -0.095*** | -0.0218 | 0.00131 | -0.00206 | -0.057*** | -0.0144 | -0.086*** | -0.0258 | -0.049*** | -0.0165 |
| SE | (0.014) | (0.016) | (0.014) | (0.013) | (0.008) | (0.002) | (0.014) | (0.011) | (0.016) | (0.015) | (0.013) | (0.018) |
| P-value | 0.0000 | 0.3070 | 0.0000 | 0.0849 | 0.8720 | 0.3650 | 0.0000 | 0.1750 | 0.0000 | 0.0960 | 0.0003 | 0.3630 |
| Constant | 0.476*** | 0.383*** | 0.365*** | 0.278*** | 0.0191*** | 0.0147*** | 0.220*** | 0.155*** | 0.424*** | 0.344*** | 0.291*** | 0.241*** |
|  | (0.025) | (0.029) | (0.025) | (0.022) | (0.005) | (0.002) | (0.014) | (0.019) | (0.022) | (0.024) | (0.016) | (0.015) |
|  |  |  |  |  |  |  |  |  |  |  |  |  |
| Observations | 2,076 | 2,110 | 2,076 | 2,110 | 2,076 | 2,110 | 2,076 | 2,110 | 2,075 | 2,107 | 2,075 | 2,107 |
| R-squared | 0.008 | 0.000 | 0.010 | 0.001 | 0.000 | 0.000 | 0.005 | 0.000 | 0.008 | 0.001 | 0.003 | 0.000 |
|  | *Below Median Pro-Charter State Policy Score* | | | | | | | | | | | |
|  | **No Signal** | **Signal** | **No Signal** | **Signal** | **No Signal** | **Signal** | **No Signal** | **Signal** | **No Signal** | **Signal** | **No Signal** | **Signal** |
| Black | -0.067*** | -0.0245 | -0.046*** | -0.0121 | 0.0171*** | 0.00449 | -0.038*** | -0.014 | -0.068*** | -0.019 | -0.0333* | -0.00266 |
| SE | (0.012) | (0.018) | (0.009) | (0.013) | (0.003) | (0.003) | (0.007) | (0.018) | (0.012) | (0.019) | (0.016) | (0.014) |
| P-value | 0.0000 | 0.1830 | 0.0000 | 0.3610 | 0.0000 | 0.1890 | 0.0000 | 0.4300 | 0.0000 | 0.3080 | 0.0396 | 0.8530 |
| Constant | 0.491*** | 0.433*** | 0.332*** | 0.291*** | 0.0170*** | 0.0269*** | 0.225*** | 0.187*** | 0.419*** | 0.368*** | 0.300*** | 0.262*** |
|  | (0.025) | (0.033) | (0.016) | (0.020) | (0.003) | (0.003) | (0.013) | (0.011) | (0.018) | (0.025) | (0.016) | (0.018) |
|  |  |  |  |  |  |  |  |  |  |  |  |  |
| Observations | 2,822 | 2,882 | 2,822 | 2,882 | 2,822 | 2,882 | 2,822 | 2,882 | 2,821 | 2,878 | 2,820 | 2,878 |
| R-squared | 0.004 | 0.001 | 0.003 | 0.000 | 0.003 | 0.000 | 0.002 | 0.000 | 0.005 | 0.000 | 0.001 | 0.000 |
| *Note: These models exclude state fixed effects because we are interested in cross-state variation in the policy regime governing charter schools. We acquired data on pro-charter policies from the National Alliance for Public Charter Schools. Robust standard errors clustered by management organization and school identifier in parentheses *** p<0.001, ** p<0.01, * p<0.05* | | | | | | | | | | | | |

| **Table S7.2** Subgroup Analysis, by Management Organization Type | | | | | | | | | | | | | |
| --- | --- | --- | --- | --- | --- | --- | --- | --- | --- | --- | --- | --- | --- |
|  | Response | Response | Answered How to Apply | Answered How to Apply | Follow Up Questions Asked | Follow Up Questions Asked | Answered Yes Anyone Can Apply | Answered Yes Anyone Can Apply | Greeting Included | Greeting Included | Salutation Included | Salutation Included |  |
|  | *CMO Charter Schools* | | | | | | | | | | | | |
|  | **No Signal** | **Signal** | **No Signal** | **Signal** | **No Signal** | **Signal** | **No Signal** | **Signal** | **No Signal** | **Signal** | **No Signal** | **Signal** |  |
| Black | -0.0575** | -0.0181 | -0.0474* | -0.0117 | -0.00199 | 0.00151 | -0.0509** | -0.028 | -0.0407 | -0.0228 | -0.0148 | -0.0224 |  |
|  | (0.027) | (0.028) | (0.024) | (0.023) | (0.008) | (0.007) | (0.020) | (0.021) | (0.028) | (0.024) | (0.024) | (0.030) |  |
| P-value | 0.0348 | 0.5210 | 0.0501 | 0.6090 | 0.8080 | 0.8310 | 0.0109 | 0.1830 | 0.1440 | 0.3430 | 0.5410 | 0.4520 |  |
| Constant | 0.424*** | 0.347*** | 0.288*** | 0.239*** | 0.0168** | 0.0155** | 0.183*** | 0.154*** | 0.380*** | 0.322*** | 0.281*** | 0.240*** |  |
|  | (0.021) | (0.025) | (0.019) | (0.018) | (0.006) | (0.005) | (0.018) | (0.018) | (0.019) | (0.023) | (0.017) | (0.020) |  |
|  |  |  |  |  |  |  |  |  |  |  |  |  |  |
| Observations | 1,075 | 1,110 | 1,075 | 1,110 | 1,075 | 1,110 | 1,075 | 1,110 | 1,075 | 1,108 | 1,075 | 1,107 |  |
| R-squared | 0.086 | 0.085 | 0.059 | 0.068 | 0.049 | 0.022 | 0.045 | 0.064 | 0.075 | 0.074 | 0.075 | 0.066 |  |
|  | *EMO Charter Schools* | | | | | | | | | | | | |
|  | **No Signal** | **Signal** | **No Signal** | **Signal** | **No Signal** | **Signal** | **No Signal** | **Signal** | **No Signal** | **Signal** | **No Signal** | **Signal** |  |
| Black | -0.0841** | 0.0464 | -0.0462 | 0.0197 | -0.00221 | 0.00369 | -0.0694** | -0.00786 | -0.0675* | 0.0289 | -0.00901 | 0.0214 |  |
|  | (0.040) | (0.048) | (0.034) | (0.040) | (0.008) | (0.010) | (0.028) | (0.030) | (0.034) | (0.037) | (0.035) | (0.035) |  |
| P-value | 0.0406 | 0.3390 | 0.1860 | 0.6230 | 0.7840 | 0.7070 | 0.0167 | 0.7960 | 0.0537 | 0.4370 | 0.7970 | 0.5390 |  |
| Constant | 0.385*** | 0.250*** | 0.281*** | 0.192*** | 0.0252* | 0.00856 | 0.185*** | 0.105*** | 0.330*** | 0.205*** | 0.195*** | 0.148*** |  |
|  | (0.029) | (0.049) | (0.023) | (0.041) | (0.010) | (0.005) | (0.018) | (0.028) | (0.024) | (0.040) | (0.023) | (0.031) |  |
|  |  |  |  |  |  |  |  |  |  |  |  |  |  |
| Observations | 583 | 582 | 583 | 582 | 583 | 582 | 583 | 582 | 583 | 581 | 583 | 581 |  |
| R-squared | 0.084 | 0.066 | 0.058 | 0.071 | 0.085 | 0.039 | 0.089 | 0.068 | 0.070 | 0.056 | 0.049 | 0.035 |  |
|  | *Freestanding Charter Schools* | | | | | | | | | | | | |
|  | **No Signal** | **Signal** | **No Signal** | **Signal** | **No Signal** | **Signal** | **No Signal** | **Signal** | **No Signal** | **Signal** | **No Signal** | **Signal** |  |
| Black | -0.0789*** | -0.0312* | -0.0779*** | -0.0244** | 0.0167*** | 0.00113 | -0.0424*** | -0.00801* | -0.091*** | -0.0289 | -0.0536*** | -0.00496 |  |
|  | (0.001) | (0.013) | (0.006) | (0.006) | (0.002) | (0.002) | (0.005) | (0.003) | (0.003) | (0.018) | (0.003) | (0.007) |  |
| P-value | 0.0000 | 0.0673 | 0.0003 | 0.0140 | 0.0006 | 0.6470 | 0.0012 | 0.0539 | 0.0000 | 0.1750 | 0.0001 | 0.4940 |  |
| Constant | 0.522*** | 0.463*** | 0.378*** | 0.319*** | 0.0169*** | 0.0256*** | 0.242*** | 0.193*** | 0.451*** | 0.398*** | 0.320*** | 0.274*** |  |
|  | (0.002) | (0.007) | (0.004) | (0.004) | (0.001) | (0.000) | (0.003) | (0.001) | (0.005) | (0.009) | (0.005) | (0.001) |  |
|  |  |  |  |  |  |  |  |  |  |  |  |  |  |
| Observations | 3,083 | 3,139 | 3,083 | 3,139 | 3,083 | 3,139 | 3,083 | 3,139 | 3,081 | 3,135 | 3,080 | 3,136 |  |
| R-squared | 0.024 | 0.032 | 0.032 | 0.026 | 0.016 | 0.019 | 0.027 | 0.025 | 0.028 | 0.037 | 0.022 | 0.028 |  |
| *Note: Data for management organization type was acquired from the National Alliance for Public Charter Schools. EMOs are for-profits, CMOs are non-profits, and freestanding schools are a stand-alone school designation. Each model includes state fixed effects to increase precision. Robust standard errors clustered by management organization in parentheses *** p<0.001, ** p<0.01, * p<0.05* | | | | | | | | | | | | | |

| **Table S8.1.** Descriptive Statistics of Each Racialized Name | | | |
| --- | --- | --- | --- |
| **Black Name** | **Education** | **White Name** | **Education** |
| Latoya Rivers | 12.10 | Katie Novak | 12.67 |
| Precious Washington | 12.10 | Katelyn Miller* | 12.80 |
| Shanice Booker | 12.11 | Holly Schroeder | 12.81 |
| Ebony Washington | 12.13 | Amy Mueller | 12.91 |
| Jazmine Jefferson | 12.18 | Abigail Smith* | 12.99 |
| Alexus Banks | 12.22 | Madeline Haas | 13.01 |
| Kiara Jackson | 12.22 | Emily Schmidt | 13.06 |
| Keisha Rivers | 12.23 | Caitlin Schneider | 13.19 |
| Deja Mosley | 12.31 | Molly Kruger | 13.20 |
| Jada Moseley | 12.37 | Claire Schwartz | 13.31 |
| **Total** | **12.20** |  | **12.99** |
| Note: The names above and education level data were gathered from the Butler and Homola (2017) article based on publicly available data accessed through this link: <https://dataverse.harvard.edu/dataset.xhtml?persistentId=doi:10.7910/DVN/LUGBL1> | | | |

| \| **Table S8.2.** Subgroup Analysis, by Socioeconomic Status of Black and White Names \| \| \| \| \| \| \| \| --- \| --- \| --- \| --- \| --- \| --- \| --- \| \|  \|  \|  \|  \|  \|  \|  \| \|  \| Response \| Answered How to Apply \| Follow Up Questions Asked \| Answered Yes Anyone Can Apply \| Greeting Included \| Salutation Included \| \|  \| *Above Average SES Name* \| \| \| \| \| \| \| **Black** \| -0.00136 \| -0.0381* \| 0.0188*** \| 0.00643 \| -0.0138 \| 0.0139 \| \| SE \| (0.017) \| (0.017) \| (0.004) \| (0.010) \| (0.017) \| (0.016) \| \| P-value \| 0.936 \| 0.027 \| 0.000 \| 0.518 \| 0.420 \| 0.375 \| \| **Signal** \| -0.0640*** \| -0.0651*** \| 0.00378 \| -0.0336** \| -0.0528*** \| -0.0278** \| \| SE \| (0.014) \| (0.013) \| (0.003) \| (0.011) \| (0.015) \| (0.010) \| \| P-value \| 0.000 \| 0.000 \| 0.229 \| 0.003 \| 0.000 \| 0.005 \| \| **Black*Signal** \| 0.0116 \| 0.0251 \| -0.00581 \| -0.00366 \| 0.0254 \| -0.00584 \| \| SE \| (0.033) \| (0.024) \| (0.004) \| (0.021) \| (0.028) \| (0.032) \| \| P-value \| 0.728 \| 0.297 \| 0.168 \| 0.864 \| 0.368 \| 0.856 \| \|  \|  \|  \|  \|  \|  \|  \| \| Constant \| 0.482*** \| 0.352*** \| 0.0137*** \| 0.210*** \| 0.411*** \| 0.288*** \| \|  \| (0.016) \| (0.017) \| (0.003) \| (0.012) \| (0.010) \| (0.010) \| \|  \|  \|  \|  \|  \|  \|  \| \| Observations \| 4,860 \| 4,860 \| 4,860 \| 4,860 \| 4,859 \| 4,859 \| \| R-squared \| 0.047 \| 0.038 \| 0.018 \| 0.03 \| 0.041 \| 0.037 \| \|  \| *Below Average SES Name* \| \| \| \| \| \| \| **Black** \| -0.142*** \| -0.0914*** \| 0.00376 \| -0.0941*** \| -0.133*** \| -0.0881*** \| \| SE \| (0.012) \| (0.011) \| (0.005) \| (0.013) \| (0.012) \| (0.011) \| \| P-value \| 0.000 \| 0.000 \| 0.474 \| 0.000 \| 0.000 \| 0.000 \| \| **Signal** \| -0.0832*** \| -0.0568*** \| 0.00601 \| -0.0649*** \| -0.0764*** \| -0.0602*** \| \| SE \| (0.015) \| (0.012) \| (0.005) \| (0.012) \| (0.011) \| (0.010) \| \| P-value \| 0.000 \| 0.000 \| 0.197 \| 0.000 \| 0.000 \| 0.000 \| \| **Black*Signal** \| 0.0940*** \| 0.0726*** \| -0.0148** \| 0.0656*** \| 0.0839*** \| 0.0660*** \| \| SE \| (0.025) \| (0.018) \| (0.005) \| (0.018) \| (0.022) \| (0.018) \| \| P-value \| 0.000 \| 0.000 \| 0.006 \| 0.000 \| 0.000 \| 0.000 \| \|  \|  \|  \|  \|  \|  \|  \| \| Constant \| 0.488*** \| 0.340*** \| 0.0214*** \| 0.235*** \| 0.432*** \| 0.304*** \| \|  \| (0.013) \| (0.011) \| (0.004) \| (0.009) \| (0.012) \| (0.009) \| \|  \|  \|  \|  \|  \|  \|  \| \| Observations \| 5,033 \| 5,033 \| 5,033 \| 5,033 \| 5,025 \| 5,024 \| \| R-squared \| 0.041 \| 0.024 \| 0.013 \| 0.032 \| 0.043 \| 0.027 \| \| Note: We measure the socioeconomic status (SES) of the names in the audit study based on data from Butler and Homola (2017). We match our names to the data Butler and Homola provide on the education level (our proxy measure of SES), which we balanced across White and Black names during the name selection process (see Table S8.1). Each model includes state fixed effects to increase precision. Robust standard errors clustered by management organization in parentheses *** p<0.001, ** p<0.01, * p<0.05 \| \| \| \| \| \| \| |
| --- | --- | --- | --- | --- | --- | --- | --- | --- | --- | --- | --- | --- | --- | --- | --- | --- | --- | --- | --- | --- | --- | --- | --- | --- | --- | --- | --- | --- | --- | --- | --- | --- | --- | --- | --- | --- | --- | --- | --- | --- | --- | --- | --- | --- | --- | --- | --- | --- | --- | --- | --- | --- | --- | --- | --- | --- | --- | --- | --- | --- | --- | --- | --- | --- | --- | --- | --- | --- | --- | --- | --- | --- | --- | --- | --- | --- | --- | --- | --- | --- | --- | --- | --- | --- | --- | --- | --- | --- | --- | --- | --- | --- | --- | --- | --- | --- | --- | --- | --- | --- | --- | --- | --- | --- | --- | --- | --- | --- | --- | --- | --- | --- | --- | --- | --- | --- | --- | --- | --- | --- | --- | --- | --- | --- | --- | --- | --- | --- | --- | --- | --- | --- | --- | --- | --- | --- | --- | --- | --- | --- | --- | --- | --- | --- | --- | --- | --- | --- | --- | --- | --- | --- | --- | --- | --- | --- | --- | --- | --- | --- | --- | --- | --- | --- | --- | --- | --- | --- | --- | --- | --- | --- | --- | --- | --- | --- | --- | --- | --- | --- | --- | --- | --- | --- | --- | --- | --- | --- | --- | --- | --- | --- | --- | --- | --- | --- | --- | --- | --- | --- | --- | --- | --- | --- | --- | --- | --- | --- | --- | --- | --- | --- | --- | --- | --- | --- | --- | --- | --- | --- | --- | --- | --- | --- | --- | --- | --- | --- | --- | --- | --- | --- | --- | --- | --- | --- | --- | --- | --- | --- | --- | --- | --- | --- | --- | --- | --- | --- | --- | --- | --- | --- |

| **Table S9.1** Average treatment effects, without fixed effects | | | | | | |
| --- | --- | --- | --- | --- | --- | --- |
|  | Response | Answered How to Apply | Follow Up Questions Asked | Answered Yes Anyone Can Apply | Greeting Included | Salutation Included |
| **Black** | -0.0754*** | -0.0668*** | 0.0103** | -0.0466*** | -0.076*** | -0.040*** |
| SE | (0.009) | (0.008) | (0.004) | (0.006) | (0.011) | (0.010) |
| P-value | 0.000 | 0.000 | 0.007 | 0.000 | 0.000 | 0.000 |
| **Signal** | -0.0735*** | -0.060*** | 0.0038 | -0.0494*** | -0.064*** | -0.044*** |
| SE | (0.011) | (0.008) | (0.003) | (0.008) | (0.010) | (0.007) |
| P-value | 0.000 | 0.000 | 0.251 | 0.000 | 0.000 | 0.000 |
| **Black*Signal** | 0.0553** | 0.0514*** | -0.00841* | 0.0336** | 0.0552** | 0.0318 |
| SE | (0.019) | (0.013) | (0.003) | (0.011) | (0.020) | (0.021) |
| P-value | 0.004 | 0.000 | 0.016 | 0.003 | 0.007 | 0.134 |
|  |  |  |  |  |  |  |
| Constant | 0.485*** | 0.346*** | 0.0179*** | 0.223*** | 0.421*** | 0.296*** |
|  | (0.023) | (0.019) | (0.002) | (0.012) | (0.018) | (0.014) |
|  |  |  |  |  |  |  |
| Observations | 9,897 | 9,897 | 9,897 | 9,897 | 9,888 | 9,887 |
| R-squared | 0.005 | 0.004 | 0.001 | 0.004 | 0.005 | 0.002 |
| Note: Robust standard errors clustered by management organization and school identifier in parentheses *** p<0.001, ** p<0.01, * p<0.05 | | | | | | |

| **Table S10.1** Subgroup Regression Results, by Signal Condition with Alternative Outcome Measure | | | | | | | |
| --- | --- | --- | --- | --- | --- | --- | --- |
|  | **Black** | **SE** | **P-Value** | **Constant** | **SE** | **N** | **R-squared** |
| **Primary Outcomes** |  |  |  |  |  |  |  |
| Response, No Signal | -0.074*** | (0.008) | 0.000 | 0.484*** | (0.015) | 4,899 | 0.035 |
| Response, Signal | -0.024 | (0.014) | 0.080 | 0.413*** | (0.019) | 4,994 | 0.041 |
| Answered How to Apply, No Signal | -0.066*** | (0.008) | 0.000 | 0.345*** | (0.013) | 4,899 | 0.027 |
| Answered How to Apply, Signal | -0.019 | (0.010) | 0.055 | 0.287*** | (0.013) | 4,994 | 0.026 |
| Follow Up Questions Asked, No Signal | 0.010** | (0.004) | 0.006 | 0.018*** | (0.002) | 4,894 | 0.009 |
| Follow Up Questions Asked, Signal | 0.001 | (0.002) | 0.685 | 0.022*** | (0.002) | 4,986 | 0.018 |
| **Secondary Outcomes** |  |  |  |  |  |  |  |
| Answered Can Anyone Apply, No Signal | -0.066*** | (0.005) | 0.000 | 0.268*** | (0.010) | 4,897 | 0.029 |
| Answered Can Anyone Apply, Signal | -0.014 | (0.010) | 0.169 | 0.203*** | (0.009) | 4,988 | 0.029 |
| Greeting Included, Signal | -0.076*** | (0.010) | 0.000 | 0.421*** | (0.011) | 4,897 | 0.034 |
| Greeting Included, No Signal | -0.024 | (0.014) | 0.085 | 0.359*** | (0.013) | 4,987 | 0.040 |
| Salutation Included, Signal | -0.039*** | (0.010) | 0.000 | 0.296*** | (0.008) | 4,896 | 0.023 |
| Salutation Included, No Signal | -0.011 | (0.013) | 0.406 | 0.254*** | (0.009) | 4,987 | 0.029 |
| Note: Each model includes state fixed effects to increase precision. Robust standard errors clustered by management organization and school identifier in parentheses *** p<0.001, ** p<0.01, * p<0.05 | | | | | | | |
